# Novel insights on remnant stomach following Roux-en-Y gastric bypass surgery based on histological evaluation and quantitative proteomics analysis

**DOI:** 10.1038/s41598-025-10114-x

**Published:** 2025-07-12

**Authors:** Carl I. W. Larson, Lars Fändriks, Anna Casselbrant, Ville Wallenius

**Affiliations:** 1 Department of Surgery, Institute of Clinical Sciences, Sahlgrenska Academy, University of Gothenburg, Gothenburg, Sweden; 2 Department of Surgery, Region Västra Götaland, Sahlgrenska University Hospital Östra, Gothenburg, Sweden; 3 Department of Surgical, Institute of Clinical Sciences at Sahlgrenska Academy, University of Gothenburg, Vita stråket 12, paviljong, vån 2, Gothenburg, 413 45 Sweden

**Keywords:** Physiology, Gastroenterology

## Abstract

Patients undergoing weight-reducing Roux-en-Y gastric bypass (RYGB) have immediate positive effects on metabolic health, including type 2 diabetes (T2D). Refeeding via the secluded stomach, either by a gastrotube or after gastro-gastric fistulation result in T2D relapse and weight regain. The stomach therefore seems to play an active role in metabolism. To explore histological and protein expression changes in the gastric mucosa before compared to after RYGB. Perioperatively, biopsies were taken in a non-paired manner from the stomach (fundus, corpus, antrum) in patients undergoing Sleeve Gastrectomy (SG) and patients > 8 months postoperatively after RYGB by balloon-enteroscopy. The included SG and RYGB patients did not display any obvious mucosal or luminal pathology during surgery or the balloon-enteroscopies. The gastric biopsies both at perioperatively and postoperatively were prepared för histological evaluation and for quantitative (comparative) non-targeted proteomics. The results were compared by Volcano plots, Principal Component Analysis and STRING functional protein association networks. Histologically the gastric mucosa looked normal in biopsies from all the different parts of the postoperative bypassed stomach with no clear differences compared to the perioperative samples. The perioperative biopsies generally contained significantly higher amounts of proteins involved in fatty acid metabolism, oxidative phosphorylation and ATP metabolic processes, citric acid cycle and the respiratory chain. Postoperative biopsies instead showed overall increased quantities of proteins associated with ribosomes, RNA-metabolic processes, the mitotic cycle and pancreatic secretion. The results provide novel insights into the mucosal proteome-changes in the secluded stomach following RYGB.

**Supplementary Information:**

The online version contains supplementary material available at 10.1038/s41598-025-10114-x.

## Introduction

Bariatric surgery remains the sole efficacious long-term intervention for patients with morbid obesity, exerting significant impacts on major metabolic comorbidities such as diabetes, hypertension, and dyslipidemia^1–3^. Among the various bariatric procedures, Roux-en-Y Gastric Bypass (RYGB) is the most prevalent in Sweden and best documented. This procedure primarily targets the proximal alimentary tract, including the stomach, duodenum, and proximal jejunum. Initially, RYGB was thought to act via restriction of food intake and by inducing mild malabsorption, thereby reducing caloric absorption. However, contemporary research has elucidated that other mechanisms play a more substantial role in its efficacy. The current hypothesis is that the main effect of RYGB surgery is altered hormonal signaling from the alimentary tract, e.g. secretion of appetite- and insulin regulating gut peptides (incretins; glucagon-like peptide (GLP-1), and glucose-dependent insulinotropic polypeptide (GIP)), which seem to be accountable for much of the observed effects^4,5^. Although it has been assumed that a more rapid distal delivery of nutrients could be responsible for the increase of incretin release (the “rapid hindgut delivery” hypothesis), there are data speaking against this and it is still unclear by which mechanism this happens^6^.

Obese patients with type 2 diabetes mellitus (T2D) going through RYGB surgery show an improved glycaemic control as early as days after surgery, when negligible weight decrease has occurred^7^. This non-weight dependent effect of bypassing the proximal alimentary tract (stomach and duodenum) on glycaemic control and incretin production (e.g. GLP-1) in response to meals, has been attributed the “foregut exclusion” hypothesis^8^. This comprises a theory of a hormonal foregut factor secreted from an unknown location in the proximal alimentary tract, that under the stimulation of ingested food leads to the inhibition of incretin hormones, e.g. GLP-1, which leads to the inhibition of meal-induced satiety and insulin effects. This factor has hypothetically been termed an anti-incretin, or decretin, and we have earlier suggested jejunal ketone production as being one such mechanism^9,10^. However, data suggest that such a mechanism might also exist in the remnant stomach or the duodenum, based on the effects of gastro-gastric fistulas or refeeding though a gastrotube, which both cause diabetes and weight relapse^11–14^.

In the present study, our objective was to investigate gastric mucosal protein expression patterns in the remnant stomach following RYGB. A central question was whether specific proteins or groups of proteins, produced postoperatively after RYGB, might contribute to the metabolic benefits associated with the surgery procedure. However, several ethical, financial, and technical problems made this almost impossible to be performed in living humans. In Sweden, uncomplicated patients with obesity are allowed to choose between two types of bariatric operative processes: Sleeve Gastrectomy (SG) or RYGB. In the current study, we chose to use biopsies from the resected stomachs of SG-patients (*perioperative* cohort) and those of balloon-enteroscopies, more than 8 months after RYGB (*postoperative* cohort). Histological evaluations of the gastric mucosa from both cohorts were compared in an ***un-paired manner***. In addition, quantitative (comparative) non-targeted proteomics were conducted on the gastric mucosal biopsies, and the results were analyzed using Volcano plots, Principal Component Analysis, and STRING functional protein association networks.

## Materials and methods

### Ethics

The study was approved by the Regional Ethical Review Board in Gothenburg, Sweden (Dnr: 2019 − 00337) and the Ethics Committee of Gothenburg University. Written informed consent from all study participants and were granted in accordance with the Declaration of Helsinki.

### Patients

This non-paired explorative cohort study consisted of perioperative samples retrieved from the resected stomach (fundus, corpus and antrum) of patients with obesity (body mass index (BMI ≥ 35)) undergoing primary laparoscopic SG. The postoperative cohort consisted of samples from the same sites in patients who had earlier (> 6 months to achieve weight stability) undergone RYGB, and that were planned for diagnostic laparoscopy and balloon-enteroscopy. These RYGB-patients were recruited based on a clinical indication, mainly chronic abdominal pain without a clear diagnosis, but were all judged normal and devoid of intestinal pathology (e.g. mucosal changes, severe adherences, luminal or anastomotic strictures). Patients were all from the same cohort of patients with their procedures performed at the Sahlgrenska University Hospital/Östra, Sweden. The surgical procedure (SG or RYGB) was chosen mainly based on the patients preference when no obvious contraindications were present judged by the surgeon, and with no systematic bias. In the SG cohort, 6 patients were recruited (BMI 43.7, mean age 41, 4 female, 1 T2D) and in the post-RYGB cohort, 6 patients were recruited (BMI 27, mean age 53, 2 female, time span RYGB surgery ≥ 8 month-8 years, 2 patients were on PPI treatment). Inclusion criteria were the same as the indications for primary obesity surgery: BMI ≥ 35 kg/m^2^ with or without associated risk factors (diabetes, hypertension), ability to understand information and to sign an informed consent. Exclusion criterion were: inability to understand information due to language barrier and any previous abdominal surgery (appendectomy allowed).

### Sample collection

Mucosal biopsies were taken from fundus, corpus, and antrum during SG from the excised stomach or by balloon-enteroscopy only in cases where no macroscopic pathological findings were made during the procedures (i.e. no mucosal inflammation, polyps, ulcers or luminal or anastomotic strictures, sign of severe adherences or other pathological findings that could explain the patients abdominal symptoms or influence the results). The endoscopic biopsies were taken using a single-balloon enteroscope with an inflatable balloon-overtube (Olympus, Hamburg, Germany) using standard biopsy forceps to retrieve mucosal samples. All patients were anaesthetized and intubated in accordance with standard protocol during the procedures after an overnight fast. Six to eight biopsies from each location were snap frozen in liquid nitrogen or chemically fixed in 4% phosphate-buffered formaldehyde immediately after surgery, ensuring their integrity for subsequent analysis. Snap-frozen tissue was then stored in a − 80 °C freezer. All samples were collected over a three-year period.

### Histology and immunohistochemistry

The chemically fixated biopsies were progressively dehydrated and embedded in paraffin. The tissue was then cut in 5 μm thick sections placed on glass slides and stained with hematoxylin-eosin. Morphometric measurement of the mucosa was carried out in the light microscope at x10 times magnification. Immunostaining was used to evaluate cell proliferation using Ki67 and enteroendocrine cells (EECs). Tissue sections were rehydrated and antigen retrieval was performed by boiling the sections for 20 min in 10mM citrate buffer (pH 6.0). For Ki-67 staining, an additional peroxidase inhibition step (Biocare Medical, CA, USA) was included, followed by blocking with 5% normal goat serum. The sections were then incubated overnight at 4 °C with primary antibodies against Ki67 (MA5-14520, Thermo Fischer Scientific, MA, USA; dilution 1:200) and chromogranin A (ab15160, Abcam, Cambridge, UK; dilution 1:200). Ki67 were visualized using the diaminobenzidine (DAB) method, and section were counterstained with Mayer´s hematoxylin. Visualization of chromogranin A was achieved through incubation with secondary Alexa Fluor™ 488 antibody for 2 h, followed by counter-staining with Hoechst before mounting with ProLong Gold anti-fade reagent (Thermo Fischer).

### Proteomics

Expression changes in the different parts of the gastric mucosa (fundus, corpus and antrum) before and after RYGB surgery were analyzed immediately upon completion of inclusion. The deep-frozen biopsies were prepared to the Global proteomics TMT tandem mass tag analysis according to the manufacturer´s instructions^15^. Quantitative (comparative) non-targeted proteomics was conducted on these samples, and the results were analyzed using Volcano plots, Principal Component Analysis, and STRING functional protein association networks. Only proteins being consistently changed in all patients and with at least a 50% change from baseline were included for further analysis.

### Statistical analysis

The log Fold Change (FC), i.e. the relative ratio of a specific protein between perioperative and postoperative samples, was calculated. Perseus (version 2.0.7.0) was used for statistical calculations and visualization. To identify differentially expressed proteins (DEPs), Welch’s t-test and Benjamin-Hochberg multiple testing were used on log2-transformed data. Proteins with an FDR < 0.05 and fold-change of at least 50% (log2 fold-change ≤ − 0.5 or ≥ 2.0) were considered as differentially expressed.

## Results

### Macroscopically normal remnant stomach post RYGB

**Fig. 1:**
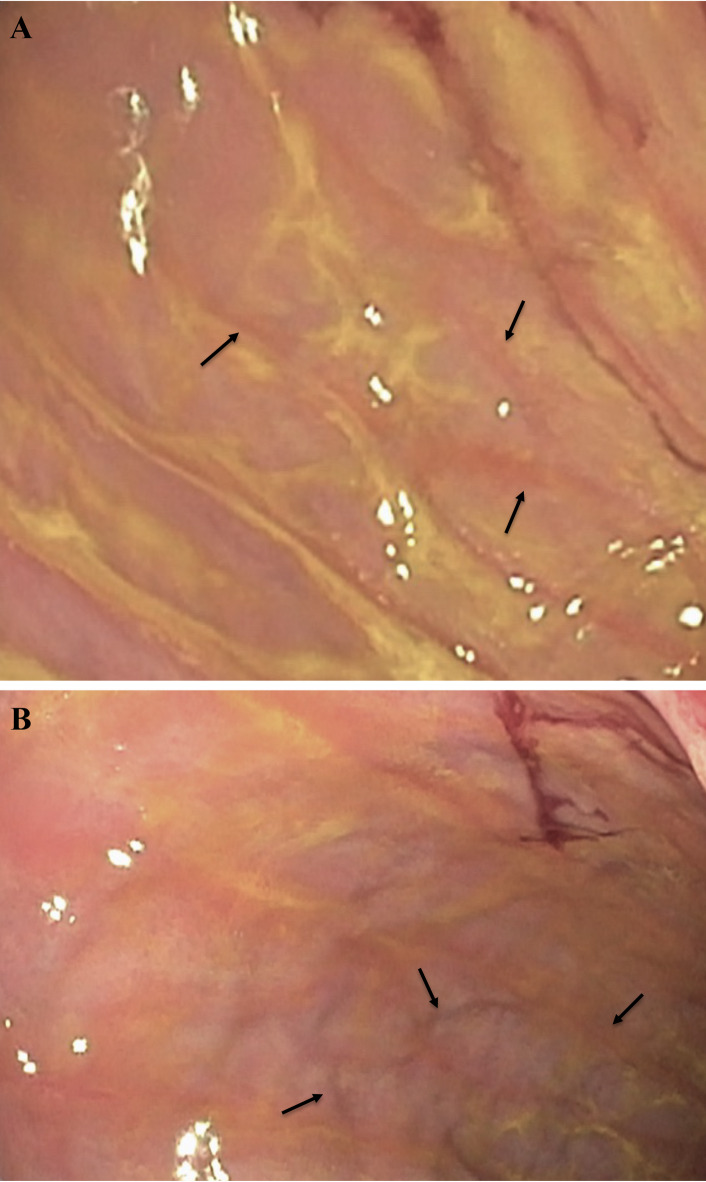
Representative endoscopic image of the remnant stomach post RYGB. The figure shows the “watermelon”-like pattern characterized by alternating bands of red and white tissue (indicated by arrows) in two different sections **A** and **B** of the antrum.

**Fig. 2:**
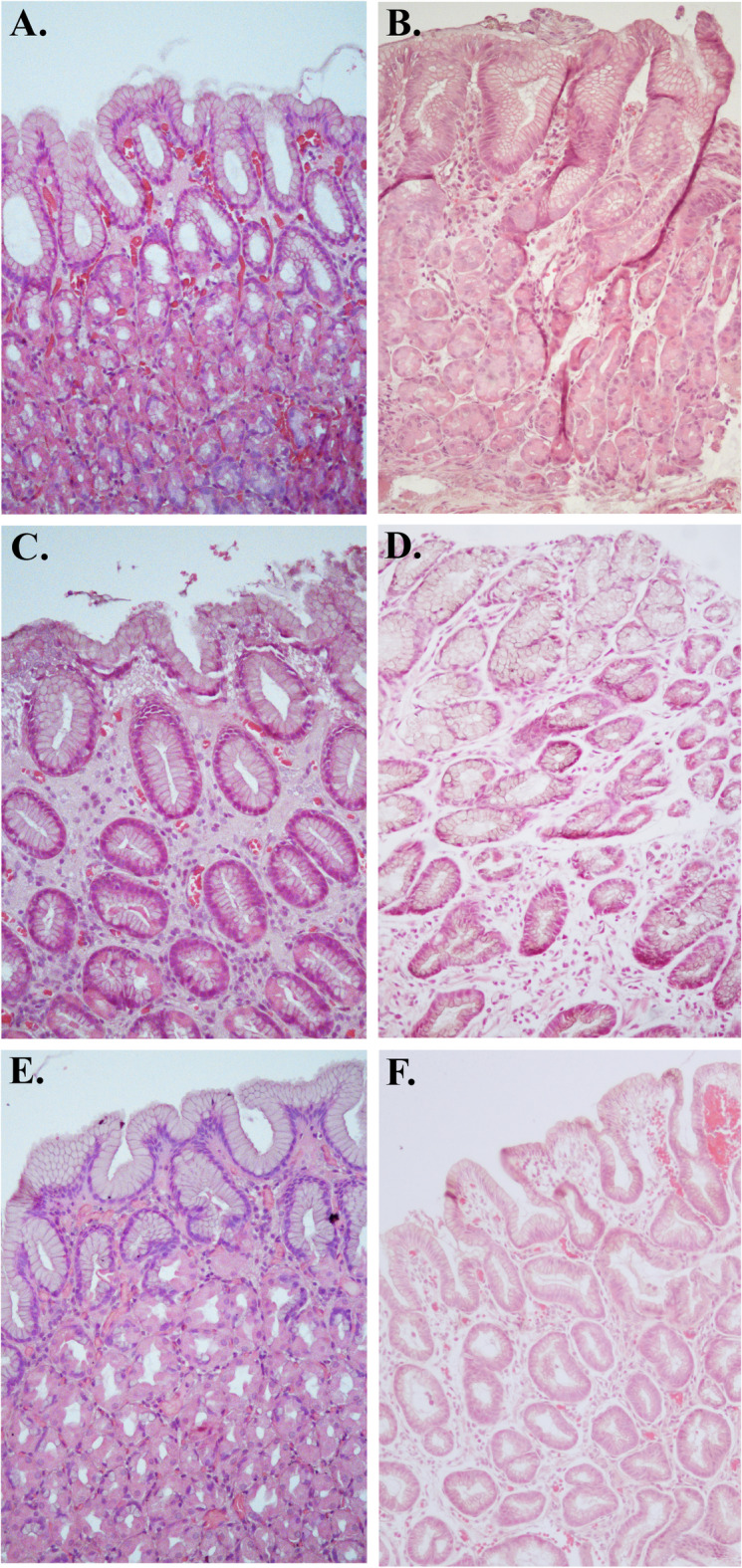
Hematoxylin-eosin-stained samples from fundus (**A**, **B**), corpus (**C**, **D**) and antrum (**E**, **F**). The human mucosal biopsies were obtained from the excised part of the stomach during sleeve gastrectomy (**A**, **C**, **E**) and in corresponding gastric bypassed mucosa ≥ 8 months post RYGB by balloon-enteroscopy (**B**, **D**, **F**).

**Fig. 3:**
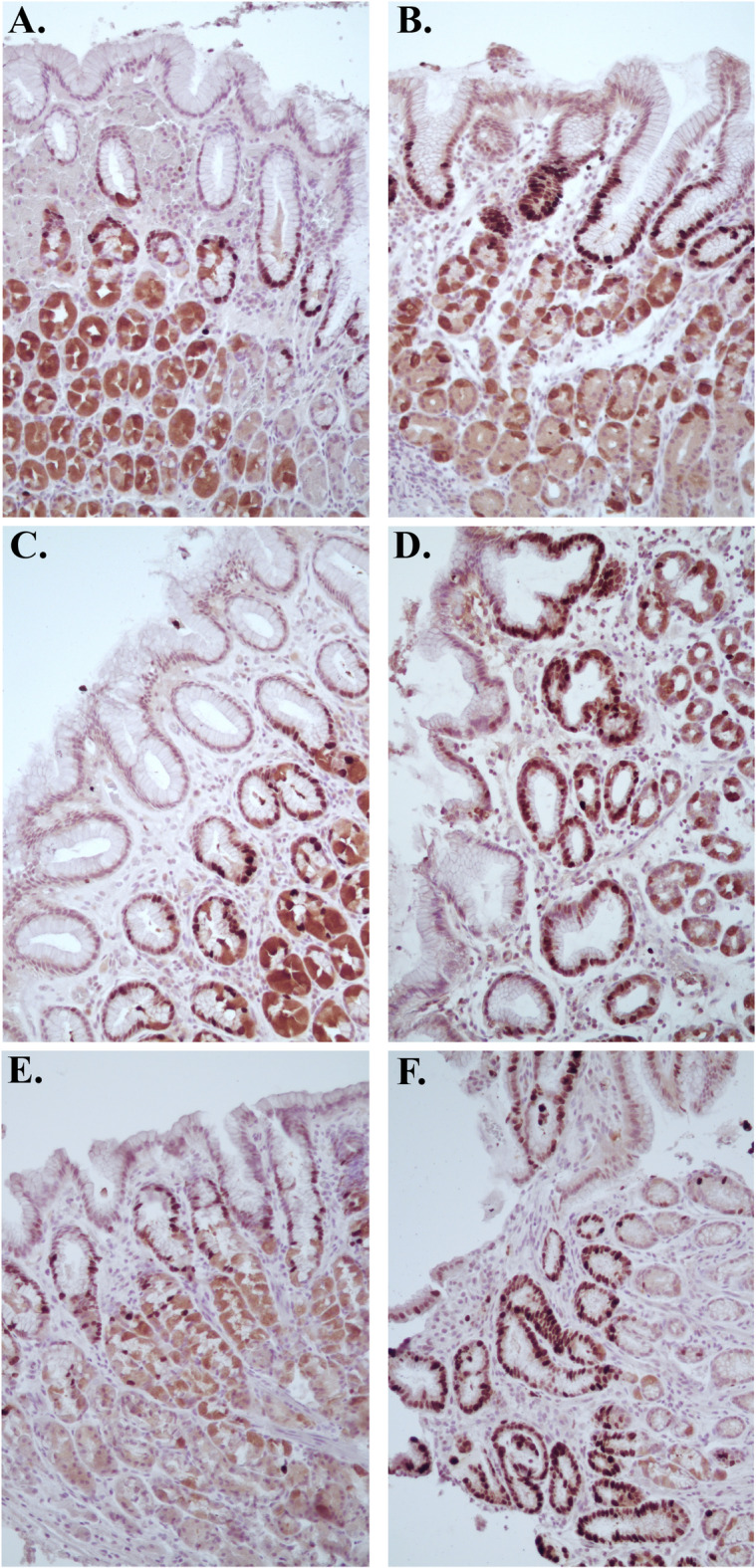
Ki67 stained samples from fundus (**A**, **B**), corpus (**C**, **D**) and antrum (**E**, **F**), obtained from the excised part of the stomach during sleeve gastrectomy (**A**, **C**, **E**) and from the corresponding gastric bypassed mucosa at ≥ 8 months post RYGB (**B**, **D**, **F**). Ki67-positive cells were predominantly localized within the mucosal glands.

**Fig. 4:**
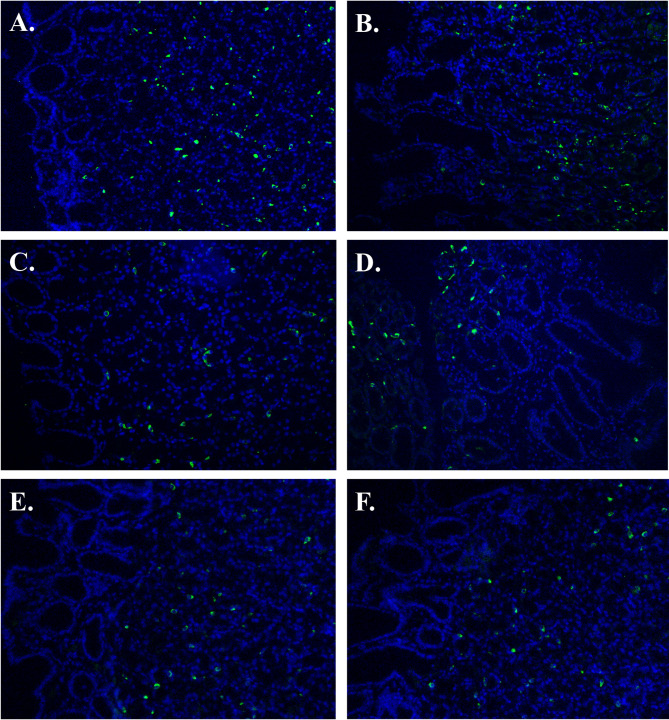
Representative immunofluorescence staining of enteroendocrine cells using the chromogranin A antibody marker in samples from the fundus (**A**, **B**), corpus (**C**, **D**) and antrum (**E**, **F**). Samples were obtained from the stomach during sleeve gastrectomy (**A**, **C**, **E**) and from the corresponding gastric bypassed mucosa at ≥ 8 months post RYGB (**B**, **D**, **F**).

The endoscopic appearance of the fundus, corpus, and antrum ≥ 8 months post-operatively gave an impression of a macroscopically somewhat different appearance compared to normal gastric mucosa, especially in the antrum, with thinner appearance of the mucosa and a “watermelon”-like pattern (Fig. 1). In contrast to this, the histological analyses revealed a mucosa that was normal in appearance (Fig. 2). The corpus glands consisted of mucous gland cells and were comprised of simple columnar epithelium where the nucleus is in the most basal part and the lumen were relatively wide in comparison to fundus glands where the glands appeared to consist of more cords of cells (chief cells) and the lumina were small. Pits and glands in the antrum also looked normal. This normal microscopic appearance of the mucosa in postoperatively samples (Fig. 2B,D,F) was very similar to the perioperatively ones (Fig. 2A,C,E). In mucosal specimens, cells exhibited immunoreactivity to Ki67, a marker of cellular proliferation. We were unable to observe any clear differences in Ki67 expression between the perioperative mucosal samples and the postoperative samples, possibly due to the limited number of participants. Most of the Ki67-labeled cells were localized within the mucosal glands (Fig. 3). The numbers of cells positive for the EECs marker chromogranin A, were abundant in both the perioperative and the postoperative mucosal samples. We were unable to ascertain any significant differences in numbers of EECs (Fig. 4).

### General protein changes

**Fig. 5:**
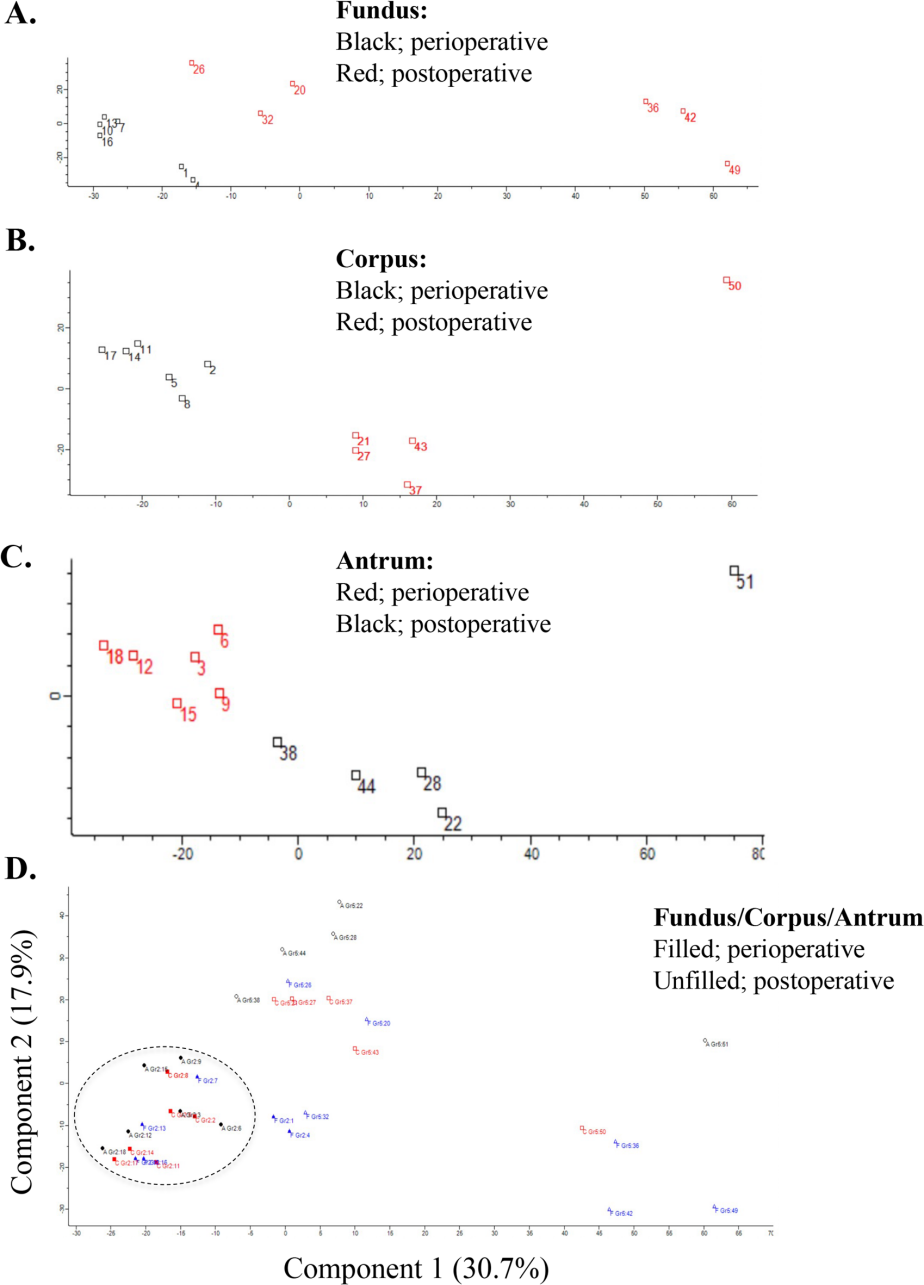
Principal component analysis (PCA) made for the proteome of all perioperative and postoperative samples from fundus (**A**), corpus (**B**) and antrum (**C**). Perioperative samples have been encircled in the Fundus/Corpus/Antrum plot (**D**). Percentages along the axes of the Fundus/Corpus/Antrum plot display the amount of variance that each axis represent. The plots illustrate that the perioperative proteomes are grouped more closely than the postoperative ones, who show more variability. Postoperatively, proteomes from the fundus show more variability when compared to the corpus and the antrum.

Intraindividual global protein expression analysis was performed to identify changes in the proteome of human mucosal fundus, corpus and antrum biopsies obtained during SG surgery, i.e. perioperative samples, and in corresponding mucosa ≥ 8 months after RYGB (postoperative samples). In general, the interindividual protein expression variability in the perioperative group of SG patients was low between individuals within respective site of gastric mucosa. In the postoperative state (RYGB) the mucosal protein expression pattern in the samples from corpus and antrum showed low variability whilst the samples from fundus displayed larger variability (Fig. 5).

Upon examining each region of the stomach (fundus, corpus and antrum), it was observed that 519 proteins exhibited an increase of $\:\ge\:50\%\:$in perioperative samples, while 255 proteins showed a similar increase of $\:\ge\:50\%\:$in postoperative samples. Specifically, the proteomics analysis identified a total of 7,929 proteins in the fundus, 7,237 proteins in the corpus, and 7,657 proteins in the antrum. Among these, significant changes were noted in 705 proteins in the fundus, 1,002 proteins in the corpus, and 1186 proteins in the antrum, with either increased or decreased amounts in perioperative compared to postoperative samples. Notably, 141 proteins (20,0%) in the fundus, 149 proteins (14,9%) in the corpus, and 212 proteins (17,9%) in the antrum exhibited more than a >2-fold increase in levels when comparing postoperative to perioperative samples (supplementary Tables 1, 2 and 3 respectively). Proteins exhibiting a greater than 2-fold decrease when comparing postoperative to perioperative levels were summarized as follows: 59 proteins (8,4%) in the fundus, 55 proteins (5,5%) in the corpus and 165 proteins (13,9%) in the antrum (supplementary Tables 1, 2 and 3 respectively).

#### Fundus

**Fig. 6:**
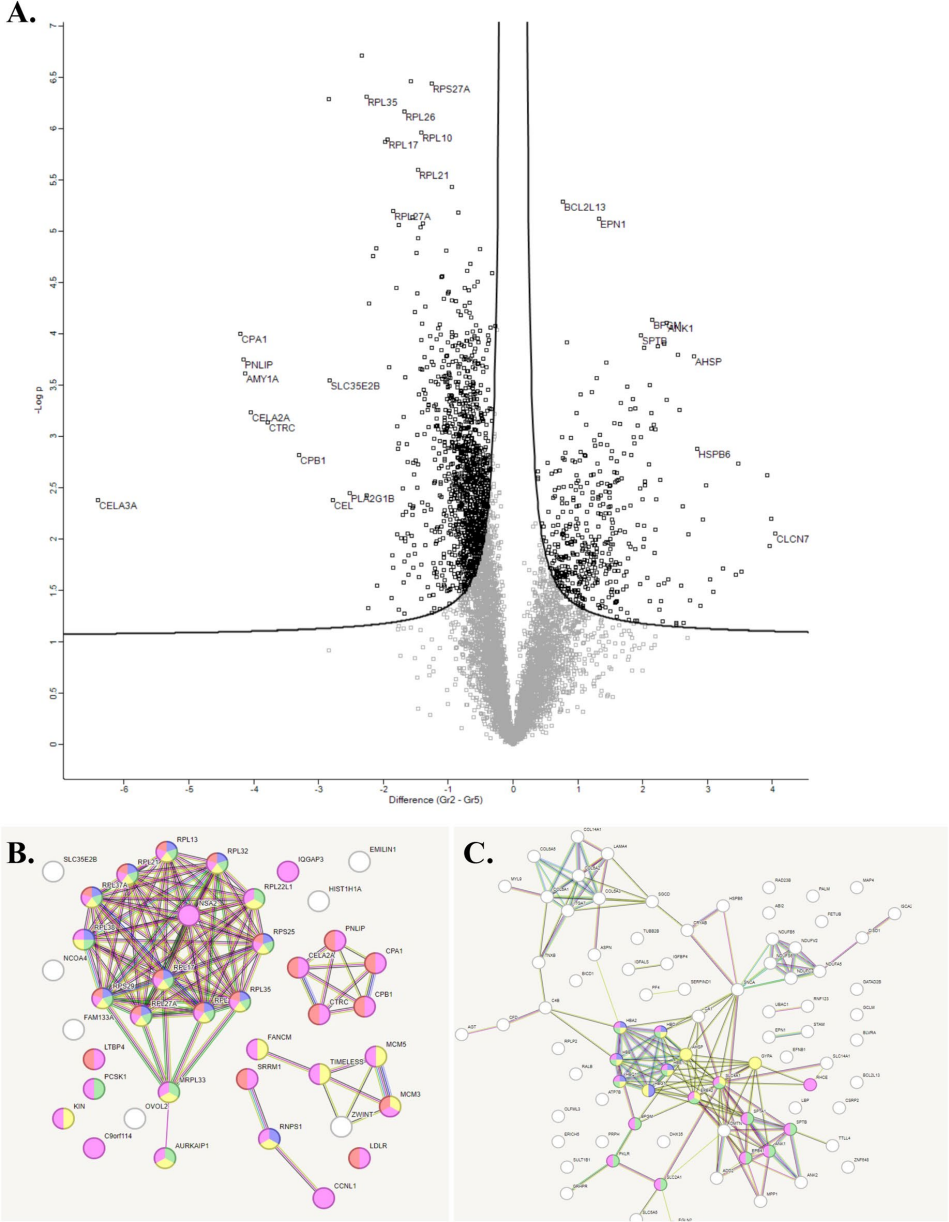
Quantitative comparative non-targeted proteomics analyses were conducted on mucosal samples from the fundus. The results are shown in Volcano plots (**A**), and STRING functional protein association networks (**B**, **C**). In the Volcano plot (**A**), the right side, along with the networks in (**C**), indicates proteins that exhibited significantly higher expression in perioperative samples compared to postoperative samples. Conversely, the left side of the Volcano plot displays proteins that showed significantly higher expression in postoperative samples relative to perioperative samples.

The volcano-plots in Fig. 6A show samples from the fundus where the right side are the most significantly upregulated proteins in the perioperative samples and the left side are the most significantly upregulated proteins postoperatively. The proteins from the volcano-plots are listed in Table 1. In the STRING analysis, the clusters of proteins are shown (Fig. 6B-C). The proteins expressed in the fundus perioperatively have differing roles in apoptosis, membrane structure and cell survival. Postoperatively, proteins mainly involved in protein synthesis, digestive enzymes and transport enzymes were upregulated. Chymotrypsin-like elastase 2 A (CELA2A) is an interesting protein (protein association networks in Fig. 6B) related to metabolic improvement that significantly increased postoperative (Table 1 and supplementary Table 1). This circulating enzyme has been described as triggering insulin secretion and degradation, as well as increasing insulin sensitivity^16^.

**Table 1:** The most regulated protein in fundus according to volcano plots.

Upregulated protein before surgery	Function of the protein	Upregulated post-surgery	Function of the protein
BCL2-Like 13 (BCL2L13)	Bcl-2 protein family involved in apoptosis	Ribosomal protein S27a (RPS27A)	Tags proteins for degradation
Epsin-1 (EPN1)	Involved in endocytosis	Ribosomal protein L35 (RPL35)	Large subunit that catalyzes protein synthesis
Bisphosphoglycerate mutase (BPGM)	Enzyme in erythrocytes, regulates hemoglobin´s oxygen affinity	Ribosomal protein L26 (RPL26)	Large subunit that catalyzes protein synthesis, part in p53 signaling
Spectrin beta chain (SPTB)	Cytoskeleton to maintain shape	Ribosomal protein L10 (RPL10)	Large subunit that catalyzes protein synthesis, negative regulation of apoptotic processes
Alpha hemoglobin stabilizing protein (AHSP)	Stabilizes alpha-hemoglobin during erythrocyte development to avoid harmful aggregation	Ribosomal protein L17 (RPL17)	Large subunit that catalyzes protein synthesis, part of the translation
Heat shock protein beta-6 (HSPB6)	Specific functions for vasodilation, platelet function, and insulin resistance	Ribosomal protein L21 (RPL21)	Large subunit that catalyzes protein synthesis, part of the translation
Chloride channel 7 alpha subunit (CLCN7)	H^+^/Cl^−^ exchange transporter	Ribosomal protein L27A (RPL27A)	Large subunit that catalyzes protein synthesis, part of the translation
Ankyrin-1 (ANK1)	Multiprotein complex in the stability and shape of the erythrocyte membrane	Carboxypeptidase A1 (CPA1)	Proteolytic enzyme, involved in protein catabolism
		Pancreatic triacylglycerol lipase (PNLIP)	Important role in fat metabolism and make fat emulsifiable
		Alpha-amylase 1 A (AMY1A)	Calcium-binding enzyme that initiates oligosaccharides digestion
		Solute carrier family 35 member E2B (SLC35E2B)	Predicted to be involved in transmembrane transport
		Chymotrypsin like elastase 2 A (CELA2A)	Circulating enzyme that trigger insulin secretion and degradation and increases insulin sensitivity
		Chymotrypsin like elastase 3 A (CELA3A)	Enzyme with digestive function, cleaves proteins, cholesterol transport and metabolism
		Chymotrypsin C (CTRC)	Regulates activation and degradation of trypsinogens and procarboxypeptidases by targeting cleavage sites
		Carboxypeptidase B1 (CPB1)	Cleave amino acids from the C-terminus of proteins and peptides
		Phospholipase A2 group 1B (PLA2G1B)	Enzyme catalyzes the release of fatty acids from glycerol-3-phosphocholines
		Carboxyl ester lipase (CEL)	Contributes to the digestion of dietary lipids

#### Corpus

**Fig. 7:**
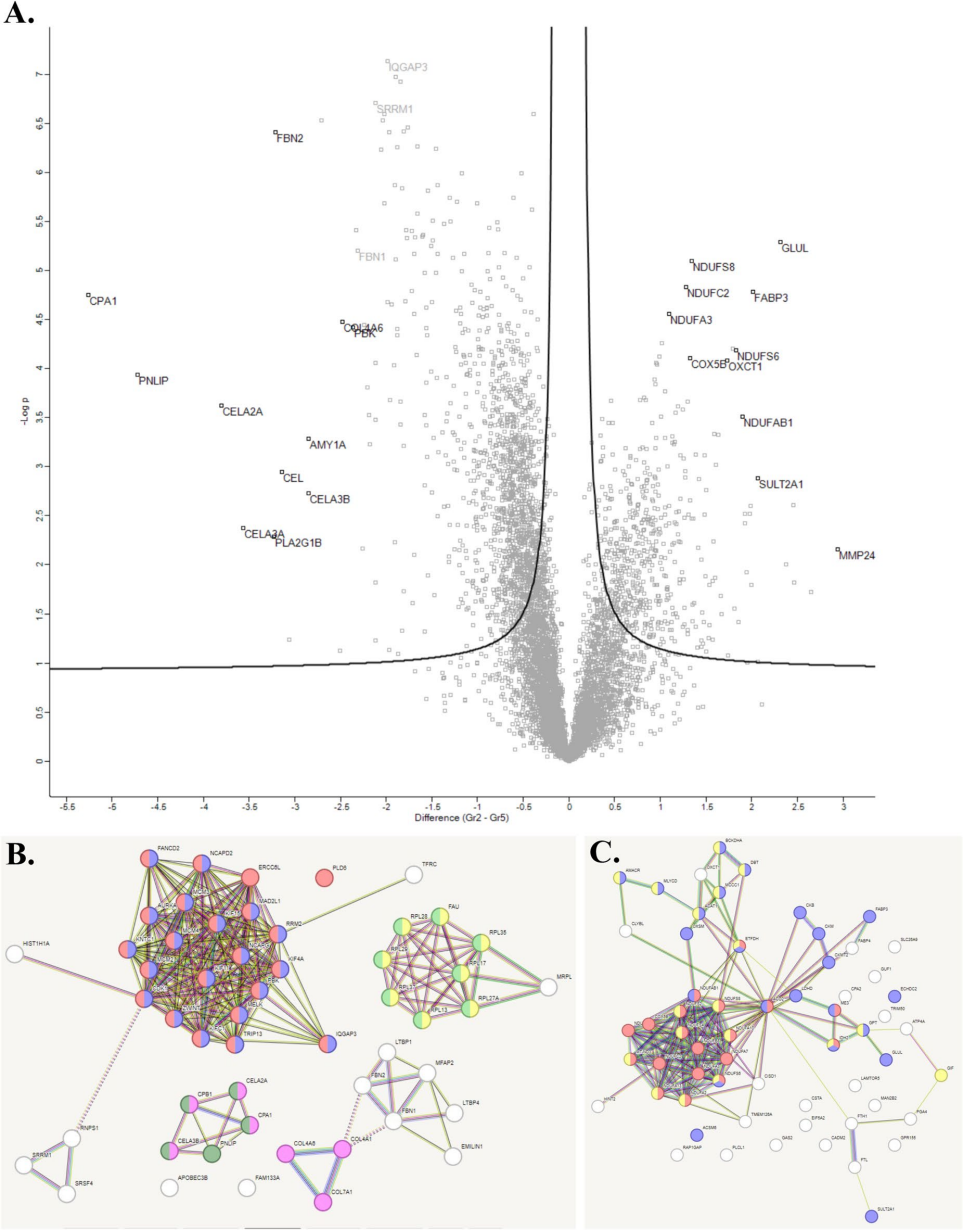
Quantitative comparative non-targeted proteomics analyses were conducted on mucosal samples from the corpus. The results are shown in Volcano plots (**A**), and STRING functional protein association networks (**B**, **C**). In the Volcano plot (**A**), the right side, along with the networks in (**C**), indicates proteins that exhibited significantly higher expression in perioperative samples compared to postoperative samples (left).

The volcano-plot and the STRING analysis are shown in Fig. 7A and B-C respectively. Regulated proteins in the volcano-plot are listed in Table 2. Proteins that were more highly expressed in the corpus mucosa perioperatively were involved in biological processes such as energy production, metabolism, detoxification, and tissue repair. One interesting finding is the protein 3-oxoacid Coa-transferase 1 (OXCT1) (networks in Fig. 7C) that was significantly more expressed before surgery (Table 2 and supplementary Table 2). This protein is a key enzyme for ketone body catabolism and is of particular interest given previous studies on intestinal ketone body formation and its role as a GLP-1 inhibitor^9^. Proteins with higher expression postoperatively were mainly involved in processes such as digestion, immune response, lipid metabolism and tissue structure.

**Table 2:** The most regulated protein in corpus according to volcano plots.

Upregulated protein before surgery	Function of the protein	Upregulated post-surgery	Function of the protein
Glutamine synthetase (GLUL)	Enzyme that catalyzes the ATP-dependent conversion of glutamate and ammonia to glutamine	Fibrillin-2 (FBN2)	Large extracellular matrix glycoprotein serves as a structural component of calcium-binding microfibrils
NADH dehydrogenease ubiquinone iron-sulfur protein 8 (NDUFS8)	Subunit, also known as Complex 1, in electron transport chain at mitochondrial inner membrane	Carboxypeptidase A1 (CPA1)	Proteolytic enzyme, involved in protein catabolism
NADH dehydrogenease ubiquinone oxidoreductase subunit C2 (NDUFC2)	The first and largest complex of the mitochondrial respiratory chain	Phospholipase A2, group 1B (PLA2G1B)	Enzyme catalyzes the release of fatty acids from glycerol-3-phosphocholines
NADH dehydrogenease ubiquinone oxidoreductase subunit A3 (NDUFA3)	Subunit in electron transport chain at mitochondrial inner membrane	Chymotrypsin-like elastase family member 3 A (CELA3A)	Enzyme with digestive function, cleaves proteins, cholesterol transport and metabolism
NADH dehydrogenease ubiquinone iron-sulfur protein 6 (NDUFS6)	First enzyme complex in the electron transport chain of mitochondria	Chymotrypsin-like elastase family member 3B (CELA3B)	Enzyme with digestive function, cleaves proteins, cholesterol transport and metabolism
NADH dehydrogenease ubiquinone oxidoreductase subunit AB1 (NDUFAB1)	Subunit in electron transport chain at mitochondrial inner membrane	Carboxyl ester lipase (CEL)	Contributes to the digestion of dietary lipids
Cytochrome c oxidase subunit 5B (COX5B)	Mitochondrial respiratory chain (ATP synthesis)	Alpha-amylase 1 (AMY1A)	First step in digestion of dietary starch and glycogen
Fatty acid-binding proteins (FABP3)	Participate in uptake, intracellular metabolism, and transport of long-chain fatty acids	Chymotrypsin like elastase 2 A (CELA2A)	Circulating enzyme that trigger insulin secretion and degradation and increases insulin sensitivity
3-oxoacid Coa-transferase 1 (OXCT1, also known as SCOT)	Key enzyme for ketone body catabolism	Pancreatic lipase (PNLIP)	Plays an important role in fat metabolism
Bile salt sulfotransferase 2A1 (SULT2A1)	Enzymes catalyze the sulfate conjugation of hormones, neurotransmitters, drugs, and xenobiotic compounds	Lymphokine-activated killer T-cell-originated protein kinase (PBK)	Enzyme of serine/threonine kinase related to mitogen-activated protein kinase (MAPKK) family
Matrix metalloproteinase 24 (MMP24)	Enzyme involved in breakdown of extracellular matrix in normal physiological processes	Collagen, type IV, alpha 6 (COL4A6)	Major structural component of basement membranes

#### Antrum

**Fig. 8:**
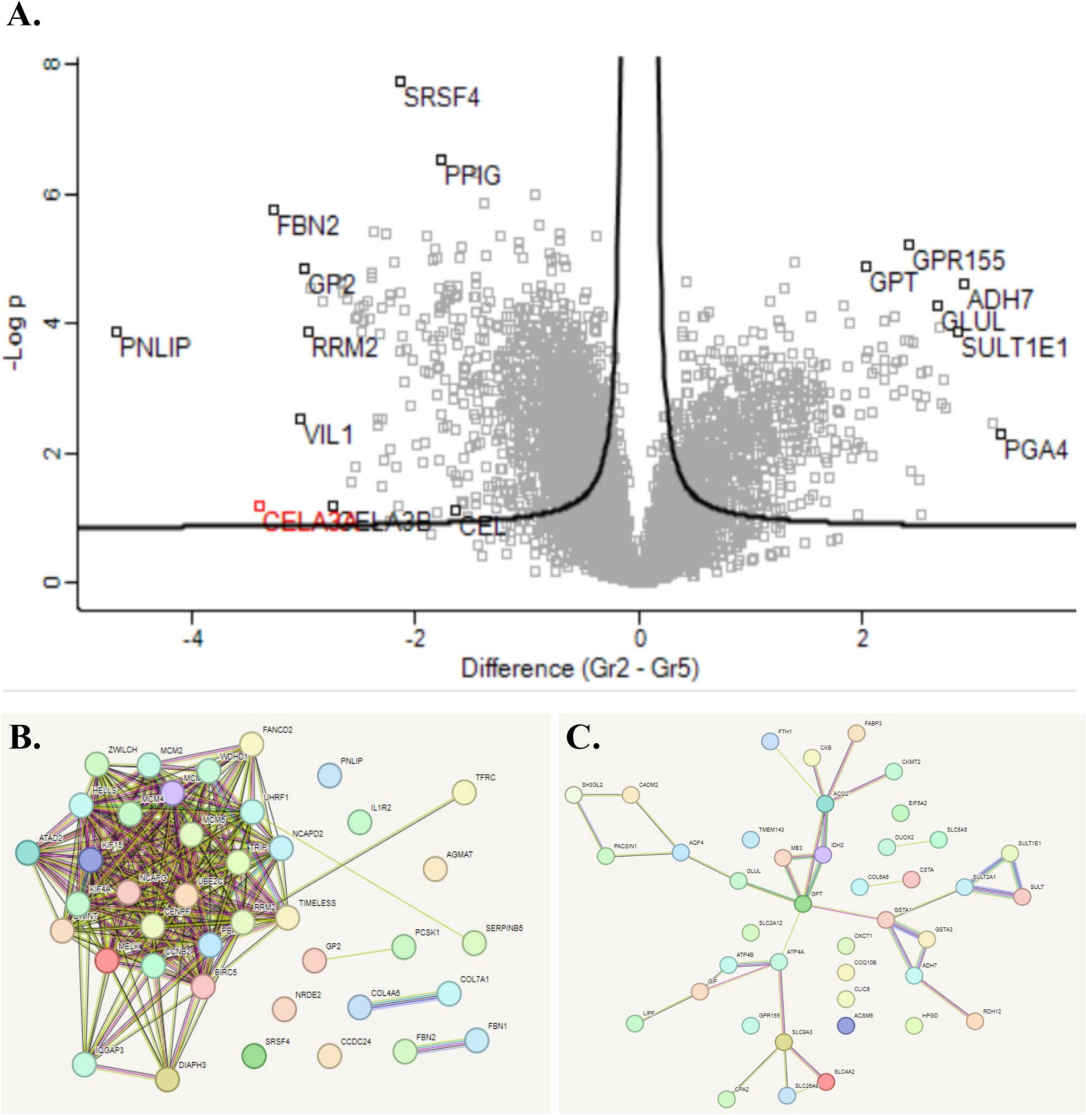
Quantitative comparative non-targeted proteomics analyses were conducted on mucosal samples from the antrum. The results are shown in Volcano plots (**A**), and STRING functional protein association networks (**B**, **C**). In the Volcano plot (**A**), the right side, along with the networks in (**C**), indicates proteins that exhibited significantly higher expression in perioperative samples compared to postoperative samples (left).

Regulated proteins from the antrum are shown in the volcano-plot and the STRING analysis in Fig. 8. and the proteins from the volcano-plot are listed in Table 3. The proteins with higher expression before surgery primarily have roles in metabolism, digestion and signaling. Postoperatively up-regulated proteins have functions in processes such as gene expression, protein folding, cell structure, metabolism and digestion.

**Table 3:** The most regulated protein in antrum according to volcano plots.

Upregulated protein before surgery	Function of the protein	Upregulated post-surgery	Function of the protein
G protein-coupled receptor 155 (GPR155)	Cholesterol-binding protein and acts as a sensor of cholesterol to signal cholesterol sufficiency	Serine and arginine rich splicing factor 4 (SRSF4)	Involved in selecting splice site before pre-mRNA
Glutamic-pyruvic transaminase (GPT)	Important regulator of glutamate levels, the major excitatory neurotransmitter of central nervous system	Peptidylprolyl isomerase G (PPIG)	Implicated in the folding, transport, and assembly of proteins
Alcohol dehydrogenase 7 (ADH7)	Catalyzes the NAD-dependent oxidation of all-trans-retinol, alcohol, and omega-hydroxy fatty acids and their derivates	Fibrillin-2 (FBN2)	Large extracellular matrix glycoprotein serves as a structural component of calcium-binding microfibrils
Glutamine synthetase (GLUL)	Enzyme that catalyzes the ATP-dependent conversion of glutamate and ammonia to glutamine	Glycoprotein 2 (GP2)	Functions as an intestinal M-cell transcytotic receptor. Internalize bacteria from lumen of the gut
Sulfotransferase family 1E member 1 (SULT1E1)	Enzyme that transfers a sulfo moiety to and from estrone, which may control levels of estrogen receptors	Pancreatic lipase (PNLIP)	Plays an important role in fat metabolism
Pepsinogen A4 (PGA4)	Precursor of the digestive enzyme pepsin digested protein, secreted by gastric chief cells	Ribonucleotide reductase regulatory subunit M2 (RRM2)	Provides the precursors necessary for DNA synthesis
		Villin 1 (VIL1)	Dominant part of the brush border cytoskeleton protein which functions in the capping, severing, and bundling of actin filaments
		Chymotrypsin-like elastase family member 3 A (CELA3A)	Enzyme with digestive function, cleaves proteins, cholesterol transport and metabolism
		Chymotrypsin-like elastase family member 3B (CELA3B)	Contributes to the digestion of dietary lipids
		Carboxyl ester lipase (CEL)	Circulating enzyme that trigger insulin secretion and degradation and increases insulin sensitivity

Overall, perioperative biopsies contained significantly higher amounts of proteins involved in fatty acid metabolism, oxidative phosphorylation, ATP metabolic processes, the citric acid cycle and the respiratory chain compared to postoperative biopsies. In contrast, postoperative biopsies from the remnant stomach showed increased quantities of proteins associated with ribosomes, RNA-metabolic processes, the mitotic cycle, and pancreatic secretion compared to perioperative biopsies (Tables 1, 2 and 3).

## Discussion

RYGB significantly alters the gastrointestinal anatomy by excluding the stomach, duodenum and part of the jejunum from nutrient flow. Consequently, bile and pancreatic juices are diverted from the proximal jejunum, leading to a modified luminal environment in the alimentary limb. It has been observed that the jejunal mucosa responds to RYGB with changes in mucosal morphology, characterized by a reduced surface epithelial area and lower, broadened villi^17^. However, it remains unclear how the protein expression pattern or the bypassed gastric mucosa changes following RYGB. The main purpose of the present study was to explore histological and protein expression changes in the gastric mucosa in subjects with obesity at baseline compared to after RYGB surgery, to thereby possibly identify mechanisms that could be linked to the metabolic effects of surgery^1–3^. The surgery leads to weight loss, but beneficial health effects on e.g. glycaemic control, occur already before any significant weight loss has occurred^7^. The mechanisms behind this are poorly understood, but the re-routing of the gastrointestinal tract likely plays a role.

Histology of the remnant stomach after RYGB has been sparsely or not at all investigated. In most cases, the remnant stomach has been examined in relation to various diagnoses, such as cancer^18,19^. Because access to the remnant stomach is difficult, it is not surprising that it is not routinely examined. All patients included in this study were referred for balloon-enteroscopy based on clinical indications. Only those patients with no obvious pathological findings during the examination were included in the study. The mucosa in the remnant stomach appeared predominantly healthy. However, in several cases, a so-called “watermelon”-like pattern characterized by alternating longitudinal redish and pale striped appearance of the mucosa was observed. This appearance gave an impression of the mucous membrane being thinner, a notion that was disproven by the histological analysis where the mucosa appeared completely normal. It may be an effect of the secluded stomach and mucosa being in a constantly coincided conformation without any periods of distension due to the absence of food passing through. Apart from the histological findings, there was no clear difference in the expression of Ki67 and EECs between perioperative and postoperative samples. The continued cell division, which apparently does not decrease in the remnant stomach, along with the abundance of EECs, indicates that the mucosa of the remnant stomach remains active and proliferates normally despite exclusion from the alimentary stream.

In the proteomics results section, we present the proteins that were significantly regulated perioperatively vs. postoperatively by location; the fundus, corpus, and antrum. However, many of the proteins were similarly regulated independent of the anatomic region of the stomach. For instance, glutamine synthetase exhibited higher expression perioperatively compared to postoperatively in the corpus and antrum. Alpha-amylase, carboxypeptidase, and phospholipase showed higher expression postoperatively in the fundus and corpus. Carboxyl esther lipase, chymotrypsin-like elastase, and pancreatic lipase were expressed at higher levels postoperatively across all three regions, the fundus, corpus, and antrum, compared to before surgery. Most similarities in protein regulation across various regions of the stomach were observed postoperatively, even though the interval since the procedure or PPI treatment ranged from 8 months to 8 years. This phenomenon could be attributed to a homogenization effect induced by surgery through several mechanisms, e.g. a such as general health improvement and decreased variability in food intake.

The only protein that was co-expressed in different regions of the stomach (corpus, antrum) before surgery was glutamine synthetase. This enzyme catalyzes the formation of glutamine from glutamate and ammonia, which is an important process for regulating ammonia levels in the body and for producing amino acids. The reduction that we observed in glutamine synthetase expression in the gastric mucosa following RYGB may reflect altered local nitrogen handling and decreased glutamine synthesis due to the ceased nutrient flow. Glutamine is a potent stimulus for GLP-1 secretion, for example, increasing release more than sevenfold in GLUTag and primary L-cell models^20^. Thus, decreased gastric glutamine synthetase could enhance glutamine availability downstream in the jejunum, promoting increased postprandial GLP-1 secretion and contributing to improved insulin sensitivity and metabolic control after surgery^21^. In general, perioperative biopsies exhibited significantly higher levels of proteins involved in fatty acid metabolism (fatty acid-binding proteins, oxoacid CoA-transferase, sulfotransferase, glutamic-pyruvic transaminase), oxidative phosphorylation and ATP metabolic processes, the citric acid cycle, and the respiratory chain (NADH dehydrogenase, cytochrome c oxidase). These changes may reflect reduced mucosal metabolic activity due to the exclusion from nutrient flow.

Postoperative biopsies instead showed overall increased quantities of proteins associated with the ribosomes (ribosomal proteins), RNA-metabolic processes (serine and arginine rich splicing factor), mitotic cycle (ribonucleotide reductase regulatory subunit), and pancreatic secretion (pancreatic lipase). The latter likely reflects increased reflux of pancreatic secretions into the secluded RYGB stomach. The upregulation of translational and mitotic markers, such as ribosomal proteins and cell cycle regulators suggests ongoing epithelial remodeling, even in the absence of nutrient exposure. Still, these changes were not reflected in increased Ki67 positivity, indicating preserved but not exaggerated epithelial turnover.

A particularly notable finding was the significant upregulation of chymotrypsin-like elastase 2 A (CELA2A) in the fundus and corpus mucosa after RYGB. CELA2A is a circulating enzyme (zymogen) that triggers insulin secretion and degradation and increases insulin sensitivity. We only found one previous study on CELA2A in humans^16^. Interestingly, the authors showed that CELA2A plasma levels increase postprandially and in parallel with insulin levels, and that inherited loss-of-function of CELA2A can cause genetic clustering of the metabolic syndrome with early onset atherosclerosis and metabolic syndrome and affect plasma insulin and platelet activation in humans^16^. As a circulating enzyme, CELA2A may have a peripheral function, but it is also locally expressed, in the pancreas, but also in the large and small intestine, liver, and white adipose tissue^16,22^. A mutated or downregulated expression of CELA2A may therefore affect insulin secretion/degradation/sensitivity and can be considered important for metabolic disease. Whether an upregulation of this protein in gastric mucosa, as we show here after RYGB, may be a contributor to beneficial metabolic effects as a consequence of bypassing the stomach needs to be determined in future studies.

Oxoacid CoA transferase 1 (OXCT1, also known as SCOT) is another interesting protein with higher expression before surgery (corpus) that converts acetoacetate to acetyl-CoA, which is an important process in ketone metabolism and energy production. As a result, OXCT1 allows cells to utilize energy stored in ketone bodies synthesized during specific conditions. We have recently identified high expression of the ketogenic enzyme mitochondrial 3-hydroxy-3-methylglutaryl-CoA synthase 2 (HMGCS2) in the jejunum of patients with obesity prior to RYGB surgery and a substantial decrease of this enzyme after surgery^9^. We have shown that the ketone bodies have an inhibitory effect on GLP-1 secretion from enteroendocrine cells in vitro^10^. We suggested that this may contribute to the increased GLP-1 levels after RYGB and have an immediate effect on the improved glucose homeostasis and improvement of T2D in the immediate phase after RYGB surgery before any weight loss has occurred^9^. Whether upregulated OXCT1 protein in the stomach can be involved in a similar way as HMGCS2 in the jejunum remains to be investigated.

One limitation of this study is the relatively small sample size, which may permit individual variations to influence the results. Nevertheless, the proteomic data presented in the results section demonstrate statistically significant changes, with expression levels exhibiting at least a twofold upregulation or downregulation. Despite this limitation, we find it justified to report these findings from the remnant stomach, due to the exceptional rarity and inaccessibility of this tissue.

In summary, to our knowledge this is the first study on the histology and protein expression pattern, conducted on the remnant secluded stomach after gastric bypass surgery. Several of the observed changes could be compatible with the improved metabolic situation after RYGB. Particularly noteworthy was the downregulation of glutamine synthase and the massive increase in the metabolically important but scarcely examined CELA2A protein expression following RYGB surgery. Future research my elucidate whether this protein plays any role in the improvement of metabolic outcomes after RYGB.

## Electronic supplementary material

Below is the link to the electronic supplementary material.

Supplementary Material 1

Supplementary Material 2

Supplementary Material 3

## Data Availability

Data availability statements: The raw proteomics data in this study have been deposited to the PRIDE database (http://www.ebi.ac.uk/pride) under accession number (PXD065508). Otherwise, please contact the submitting author to request data from the study.

## References

[1] Purnell, J. Q., Dewey, E. N. & Laferrére, B. Diabetes remission status during seven-year follow-up of the longitudinal assessment of bariatric surgery study. *J. Clin. Endocrinol. Metab.* **106**, 774–788 (2021). 33270130 10.1210/clinem/dgaa849 PMC7947785

[2] Carranza-Leon, B. G., Puzziferri, N., Adams-Huet, B., Jabbour, I. & Lingvay, I. Metabolic response 4 years after gastric bypass in a complete cohort with type 2 diabetes mellitus. *Diabetes Res. Clin. Pract.* **137**, 224–230 (2018). 29355650 10.1016/j.diabres.2017.11.022

[3] Cummings, D. E. Cohen RV. Bariatric/Metabolic surgery to treat type 2 diabetes in patients with a BMI < 35 kg/m2. *Diabetes Care* 2016:39;924– 33. 10.2337/dc16-0350 PMC4878219 27222550

[4] Moran, T. H. & Dailey, M. J. Intestinal feedback signaling and satiety. *Physiol. Behav.* **105**, 77–81 (2011). 21315751 10.1016/j.physbeh.2011.02.005 PMC3143258

[5] Goldstone, A. P., Miras, A. D. & Scholtz, S. Link between increased satiety gut hormones and reduced food reward after gastric bypass surgery for obesity. *J. Clin. Endocrinol. Metab.* **101**, 599–609 (2016). 26580235 10.1210/jc.2015-2665 PMC4880130

[6] Miras, A. D., Kamocka, A. & Pérez-Pevida, B. The effect of standard versus longer intestinal bypass on GLP-1 regulation and glucose metabolism in patients with type 2 diabetes undergoing roux-en-Y gastric bypass: the long-limb study. *Diabetes Care*. **44**, 1082–1090 (2021). 33158945 10.2337/dc20-0762 PMC8132320

[7] Wallenius, V. et al. Glycemic control after sleeve gastrectomy and Roux-En-Y gastric bypass in obese subjects with type 2 diabetes mellitus. *Obes. Surg.* **28**, 1461–1472 (2018). 29264780 10.1007/s11695-017-3061-3 PMC5973990

[8] Rubino, F., Forgione, A. & Cummings, D. E. The mechanism of diabetes control after Gastrointestinal bypass surgery reveals a role of the proximal small intestine in the pathophysiology of type 2 diabetes. *Ann. Surg.* **244**, 741–749 (2006). 17060767 10.1097/01.sla.0000224726.61448.1b PMC1856597

[9] Wallenius, V., Elias, E. & Elebring, E. Suppression of enteroendocrine cell glucagon-like peptide (GLP)-1 release by fat-induced small intestional ketogenesis: a mechanism targeted by Roux-en-Y gastric bypass surgery but not by preoperative very-low-calorie diet. *Gut* **69**, 1423–1431 (2019). 31753852 10.1136/gutjnl-2019-319372 PMC7347417

[10] Elebring, E., Casselbrant, A., Persson, S. M. T., Fändriks, L. & Wallenius, V. BHB inhibits glucose-induced GLP-1 secretion in GLUTag and human jejunal enteroids. *J. Mol. Endocrinol.* **70**, e220115 (2023). 36810364 10.1530/JME-22-0115

[11] Meister, K. M., Schauer, P. R., Brethauer, S. A. & Aminian, A. Effect of gastrogastric fistula closure in type 2 diabetes. *Obes. Surg.* **28**, 1086–1090 (2018). 29090378 10.1007/s11695-017-2976-z

[12] Jirapinyo, P., Thompson, A. C., Kröner, P. T., Chan, W. W. & Thompson, C. C. Metabolic effect of foregut exclusion demonstrated by the impact of gastrogastric fistula on recurrence of diabetes. *J. Am. Coll. Surg.* **226**, 259–266 (2018). 29274838 10.1016/j.jamcollsurg.2017.12.015 PMC5826850

[13] Pina, L. et al. Bariatric revisional surgery for gastrogastric fistula following Roux-en-Y gastric bypass positively impacts weight loss. *Surg. Obes. Relat. Dis.* **19**, 626–631 (2023). 36646542 10.1016/j.soard.2022.12.022

[14] Lindqvist, A., Spégel, P. & Ekelund, M. Effects of ingestion routes on hormonal and metabolic profiles in gastric-bypassed humans. *J. Clin. Endocrinol. Metab.* **98**, E856–E861 (2013). 23633201 10.1210/jc.2012-3996

[15] Wiśniewski, J. R., Zougman, A., Nagaraj, N. & Mann, M. Universal sample Preparation method for proteome analysis. *Nat. Methods*. **6**, 359–362 (2009). 19377485 10.1038/nmeth.1322

[16] Esteghamat, F., Broughton, J. S. & Smith, E. CELA2A mutations predispose to early-onset atherosclerosis and metabolic syndrome and affect plasma insulin and platelet activation. *Nat. Genet.* **51**, 1233–1243 (2019). 31358993 10.1038/s41588-019-0470-3 PMC6675645

[17] Spak, E., Björklund, P. & Helander, H. F. Changes in the mucosa of the Roux-limb after gastric bypass surgery. *Histopathology* **57**, 680–688 (2010). 21054493 10.1111/j.1365-2559.2010.03677.x

[18] Tornese, S., Aiolfi, A. & Bonitta, G. Remnant gastric cancer after Roux-en-Y gastric bypass: narrative review of the literature. *Obes. Syrgery*. **29**, 2609–2613 (2019). 10.1007/s11695-019-03892-7 31001760

[19] Pandya, S. R., Kenney, L. M. & Hughes, M. S. Gatrointesinal stromal tumor in the excluded gastric remnant after Roux-en-Y gastric bypass. *Am. Surg.* **89**, 3311–3312 (2023). 36866534 10.1177/00031348231160839

[20] Reimann, F., Williams, L., da Silva Xavier, G., Rutter, G. A. & Gribble, F. M. Glutamine potently stimulates glucagon-like peptide-1 secretion from GLUTag cells. *Diabetologia* **47** (9), 1592–1601 (2004). 15365617 10.1007/s00125-004-1498-0

[21] Gwen Tolhurst, Y. et al. Glutamine triggers and potentiates glucagon-like peptide-1 secretion by Raising cytosolic Ca2 + and cAMP. *Endocrinology* **152** (2), 405–413 (2011). 21209017 10.1210/en.2010-0956 PMC3140224

[22] Motta, J. P., Rolland, C. & Edir, A. Epithelial production of elastase is increased in inflammatory bowel disease and causes mucosal inflammation. *Mucosal Immunol.* **14**, 667–678 (2021). 33674762 10.1038/s41385-021-00375-w PMC8075934

