# Supplementary material for: Novel insights on remnant stomach following Roux-en-Y gastric bypass surgery based on histological evaluation and quantitative proteomics analysis
Source: Sci Rep. 2025 Jul 12;15:25243. doi: 10.1038/s41598-025-10114-x (PMC12255712; doi:10.1038/s41598-025-10114-x)
Supplement: Supplementary file 1 — Supplementary Material 1 [file 41598_2025_10114_MOESM1_ESM.docx]

**Table 1.** List of protein expression in the fundus displaying a fold change greater than 2 (indicating a doubled increase postoperative) or a 2-fold decrease (indicating a 50% decrease perioperative).

| ***Accession Symbol Fold Change Description***  ***Upregulated protein expression*** | | | |
| --- | --- | --- | --- |
| P08217 | CELA2A | 30.56 | Chymotrypsin-like elastase family member 2A |
| P16233 | PNLIP | 30.29 | Pancreatic triacylglycerol lipase |
| P0DUB6 | AMY1A | 29.03 | Alpha-amylase 1A |
| Q99895 | CTRC | 27.41 | Chymotrypsin-C |
| P15085 | CPA1 | 27.14 | Carboxypeptidase A1 |
| P15086 | CPB1 | 12.0 | Carboxypeptidase B |
| P0CK96 | SLC35E2B | 9.45 | Solute carrier family 35 member E2B |
| Q8N9E0 | FAM133A | 7.10 | Protein FAM133A |
| Q9NWT8 | AURKAIP1 | 5.02 | Aurora kinase A-interacting protein |
| P42766 | RPL35 | 4.96 | 60S ribosomal protein L35 |
| Q5T280 | SPOUT1 | 4.60 | Putative methyltransferase C9orf114 |
| O95478 | NSA2 | 4.60 | Ribosome biogenesis protein NSA2 homolog |
| O60870 | KIN | 4.50 | DNA/RNA-binding protein KIN17 |
| Q15287 | RNPS1 | 3.97 | RNA-binding protein with serine-rich domain 1 |
| P18621 | RPL17 | 3.82 | 60S ribosomal protein L17 |
| P46776 | RPL27A | 3.7 | 60S ribosomal protein L27a |
| O95229 | ZWINT | 3.60 | ZW10 interactor |
| Q6P5R6 | RPL22L1 | 3.59 | 60S ribosomal protein L22-like 1 |
| Q02539 | H1-1 | 3.59 | Histone H1.1 |
| P62910 | RPL32 | 3.46 | 60S ribosomal protein L32 |
| Q9UNS1 | TIMELESS | 3.44 | Protein timeless homolog |
| P26373 | RPL13 | 3.40 | 60S ribosomal protein L13 |
| Q86VI3 | IQGAP3 | 3.37 | Ras GTPase-activating-like protein IQGAP3 |
| Q9UK58 | CCNL1 | 3.36 | Cyclin-L1 |
| P29120 | PCSK1 | 3.33 | Neuroendocrine convertase 1 |
| P61513 | RPL37A | 3.28 | 60S ribosomal protein L37a |
| P25205 | MCM3 | 3.17 | DNA replication licensing factor MCM3 |
| P25205 | RPL26 | 3.16 | 60S ribosomal protein L26 |
| P01130 | LDLR | 3.04 | Low-density lipoprotein receptor |
| P01130 | MRPL33 | 3.03 | 39S ribosomal protein L33, mitochondrial |
| P33992 | MCM5 | 2.99 | DNA replication licensing factor MCM5 |
| Q8IYB3 | SRRM1 | 2.96 | Serine/arginine repetitive matrix protein 1 |
| Q8IYB3 | RPS25 | 2.93 | 40S ribosomal protein S25 |
| Q8N2S1 | LTBP4 | 2.87 | Latent-transforming growth factor beta-binding protein 4 |
| Q13772 | NCOA4 | 2.84 | Nuclear receptor coactivator 4 |
| Q13772 | RPS29 | 2.84 | 40S ribosomal protein S29 |
| Q13772 | RPL38 | 2.82 | 60S ribosomal protein L38 |
| P46778 | RPL21 | 2.82 | 60S ribosomal protein L21 |
| P46778 | EMILIN1 | 2.82 | EMILIN-1 |
| Q8IYD8 | FANCM | 2.80 | Fanconi anemia group M protein |
| Q8IYD8 | OVOL2 | 2.77 | Transcription factor Ovo-like 2 |
| Q8IYD8 | LAMB3 | 2.76 | Laminin subunit beta-3 |
| Q6UWR7 | ENPP6 | 2.74 | Glycerophosphocholine cholinephosphodiesterase ENPP6 |
| P62269 | RPS18 | 2.71 | 40S ribosomal protein S18 |
| Q13257 | MAD2L1 | 2.69 | Mitotic spindle assembly checkpoint protein MAD2A |
| Q6DKI1 | RPL7L1 | 2.68 | 60S ribosomal protein L7-like 1 |
| P27635 | RPL10 | 2.67 | 60S ribosomal protein L10 |
| Q9BW85 | YJU2 | 2.66 | Splicing factor YJU2 |
| Q86VM9 | ZC3H18 | 2.65 | Zinc finger CCCH domain-containing protein 18 |
| P62266 | RPS23 | 2.64 | 40S ribosomal protein S23 |
| P16401 | H1-5 | 2.64 | Histone H1.5 |
| P16401 | RPS20 | 2.61 | 40S ribosomal protein S20 |
| Q9Y6R7 | FCGBP | 2.60 | IgGFc-binding protein |
| P08708 | RPS17 | 2.60 | 40S ribosomal protein S17 |
| Q8IYS1 | PM20D2 | 2.59 | Xaa-Arg dipeptidase |
| P46779 | RPL28 | 2.58 | 60S ribosomal protein L28 |
| Q16787 | LAMA3 | 2.58 | Laminin subunit alpha-3 |
| O14578 | CIT | 2.56 | Citron Rho-interacting kinase |
| O94772 | LY6H | 2.53 | Citron Rho-interacting kinase |
| O94772 | RPL24 | 2.49 | 60S ribosomal protein L24 |
| P56182 | RRP1 | 2.48 | 60S ribosomal protein L24 |
| Q15021 | NCAPD2 | 2.45 | Condensin complex subunit 1 |
| Q9BQE9 | BCL7B | 2.44 | Condensin complex subunit 1 |
| Q96EP1 | CHFR | 2.42 | E3 ubiquitin-protein ligase CHFR |
| P62753 | RPS6 | 2.41 | 40S ribosomal protein S6 |
| Q9NRG1 | PRTFDC1 | 2.40 | Phosphoribosyltransferase domain-containing protein 1 |
| Q9NRG1 | PRPF38A | 2.39 | Pre-mRNA-splicing factor 38A |
| P35269 | GTF2F1 | 2.39 | General transcription factor IIF subunit 1 |
| P49207 | RPL34 | 2.39 | 60S ribosomal protein L34 |
| P62979 | RPS27A | 2.38 | Ubiquitin-40S ribosomal protein S27a |
| P50748 | KNTC1 | 2.36 | Kinetochore-associated protein 1 |
| P78314 | SH3BP2 | 2.32 | SH3 domain-binding protein 2 |
| Q9BSV6 | TSEN34 | 2.32 | tRNA-splicing endonuclease subunit Sen34 |
| O96017 | CHEK2 | 2.30 | Serine/threonine-protein kinase Chk2 |
| P61964 | WDR5 | 2.29 | WD repeat-containing protein 5 |
| P84098 | RPL19 | 2.29 | 60S ribosomal protein L19 |
| Q9Y5Y4 | PTGDR2 | 2.29 | Prostaglandin D2 receptor 2 |
| P62847 | RPS24 | 2.28 | 40S ribosomal protein S24 |
| P48436 | SOX9 | 2.28 | Transcription factor SOX-9 |
| P40429 | RPL13A | 2.27 | 60S ribosomal protein L13a |
| Q9H773 | DCTPP1 | 2.27 | dCTP pyrophosphatase 1 |
| P46087 | NOP2 | 2.25 | Probable 28S rRNA (cytosine(4447)-C(5))-methyltransferase |
| O00541 | PES1 | 2.22 | Pescadillo homolog |
| Q14766 | LTBP1 | 2.22 | Latent-transforming growth factor beta-binding protein 1 |
| Q9Y221 | NIP7 | 2.22 | 60S ribosome subunit biogenesis protein NIP7 homolog |
| Q92979 | EMG1 | 2.21 | Ribosomal RNA small subunit methyltransferase NEP1 |
| Q9GZL7 | WDR12 | 2.21 | Ribosome biogenesis protein WDR12 |
| Q9Y3B9 | RRP15 | 2.21 | RRP15-like protein |
| Q15910 | EZH2 | 2.19 | Histone-lysine N-methyltransferase EZH2 |
| P14678 | SNRPB | 2.19 | Small nuclear ribonucleoprotein-associated proteins B and B' |
| Q9NNW5 | WDR6 | 2.18 | WD repeat-containing protein 6 |
| Q15397 | PUM3 | 2.18 | Pumilio homolog 3 |
| O43688 | PLPP2 | 2.17 | Phospholipid phosphatase 2 |
| P62314 | SNRPD1 | 2.17 | Small nuclear ribonucleoprotein Sm D1 |
| O43818 | RRP9 | 2.17 | U3 small nucleolar RNA-interacting protein 2 |
| Q96IK1 | BOD1 | 2.17 | Biorientation of chromosomes in cell division protein 1 |
| P0C2W1 | FBXO45 | 2.15 | F-box/SPRY domain-containing protein 1 |
| Q9NRX1 | PNO1 | 2.14 | RNA-binding protein PNO1 |
| Q9NXF7 | DCAF16 | 2.13 | DDB1- and CUL4-associated factor 16 |
| O95059 | RPP14 | 2.13 | Ribonuclease P protein subunit p14 |
| Q96EZ8 | MCRS1 | 2.12 | Microspherule protein 1 OS=Homo sapiens |
| Q86UY8 | NT5DC3 | 2.12 | 5'-nucleotidase domain-containing protein 3 |
| Q9Y6I8 | PXMP4 | 2.12 | Peroxisomal membrane protein 4 |
| Q6PD62 | CTR9 | 2.12 | RNA polymerase-associated protein CTR9 homolog |
| P78504 | JAG1 | 2.12 | Protein jagged-1 |
| Q8NEF9 | SRFBP1 | 2.12 | Serum response factor-binding protein 1 |
| Q13601 | KRR1 | 2.12 | KRR1 small subunit processome component homolog |
| Q6P1L8 | MRPL14 | 2.11 | 39S ribosomal protein L14, mitochondrial |
| Q8WWQ0 | PHIP | 2.11 | PH-interacting protein |
| P78345 | RPP38 | 2.10 | Ribonuclease P protein subunit p38 |
| Q8NHQ9 | DDX55 | 2.10 | ATP-dependent RNA helicase DDX55 |
| P06727 | APOA4 | 2.10 | Apolipoprotein A-IV |
| O60287 | URB1 | 2.10 | Nucleolar pre-ribosomal-associated protein 1 |
| P41223 | BUD31 | 2.08 | Protein BUD31 homolog |
| O94762 | RECQL5 | 2.08 | ATP-dependent DNA helicase Q5 |
| O00566 | MPHOSPH10 | 2.08 | U3 small nucleolar ribonucleoprotein protein MPP10 |
| Q76FK4 | NOL8 | 2.08 | Nucleolar protein 8 |
| Q92820 | GGH | 2.08 | Gamma-glutamyl hydrolase |
| Q8N5V2 | NGEF | 2.08 | Ephexin-1 |
| Q9UPN6 | SCAF8 | 2.07 | SR-related and CTD-associated factor 8 |
| Q13895 | BYSL | 2.07 | Bystin |
| O43291 | SPINT2 | 2.07 | Kunitz-type protease inhibitor 2 |
| Q8N5M4 | TTC9C | 2.06 | Tetratricopeptide repeat protein 9C |
| Q15652 | JMJD1C | 2.05 | Probable JmjC domain-containing histone demethylation protein 2C |
| Q6ZTQ3 | RASSF6 | 2.05 | Ras association domain-containing protein 6 |
| P52701 | MSH6 | 2.05 | DNA mismatch repair protein Msh6 |
| Q29RF7 | PDS5A | 2.05 | Sister chromatid cohesion protein PDS5 homolog A |
| O60678 | PRMT3 | 2.04 | Protein arginine N-methyltransferase 3 |
| Q9NWT1 | PAK1IP1 | 2.04 | p21-activated protein kinase-interacting protein 1 |
| Q9Y3T9 | NOC2L | 2.04 | Nucleolar complex protein 2 homolog |
| Q15154 | PCM1 | 2.03 | Pericentriolar material 1 protein |
| O75764 | TCEA3 | 2.03 | Transcription elongation factor A protein 3 |
| P36578 | RPL4 | 2.03 | 60S ribosomal protein L4 |
| Q03701 | CEBPZ | 2.03 | CCAAT/enhancer-binding protein zeta |
| Q5C9Z4 | NOM1 | 2.02 | Nucleolar MIF4G domain-containing protein 1 |
| Q9NVU7 | SDAD1 | 2.02 | Protein SDA1 homolog |
| Q9H6D7 | HAUS4 | 2.01 | HAUS augmin-like complex subunit 4 |
| Q96QC0 | PPP1R10 | 2.01 | Serine/threonine-protein phosphatase 1 regulatory subunit 10 |
| Q8N806 | UBR7 | 2.01 | Putative E3 ubiquitin-protein ligase UBR7 |
| Q16875 | PFKFB3 | 2.00 | 6-phosphofructo-2-kinase/fructose-2,6-bisphosphatase 3 |
| P34896 | SHMT1 | 2.00 | Serine hydroxymethyltransferase, cytosolic |
| ***Accession Symbol Fold Change Description***  ***Downregulated protein expression*** | | | |
| Q9NZD4 | AHSP | 0.15 | Alpha-hemoglobin-stabilizing protein |
| Q96G01 | BICD1 | 0.17 | Protein bicaudal D homolog 1 |
| P02724 | GYPA | 0.19 | Glycophorin-A |
| P16157 | ANK1 | 0.19 | Ankyrin-1 |
| P16452 | EPB42 | 0.19 | Protein 4.2 |
| P09105 | HBQ1 | 0.20 | Hemoglobin subunit theta-1 |
| P02730 | SLC4A1 | 0.21 | Band 3 anion transport protein |
| P37840 | SNCA | 0.21 | Alpha-synuclein |
| P18577 | RHCE | 0.22 | Blood group Rh(CE) polypeptide |
| Q14679 | TTLL4 | 0.22 | Tubulin monoglutamylase TTLL4 |
| Q9H5Z1 | DHX35 | 0.22 | Probable ATP-dependent RNA helicase DHX35 |
| P02549 | SPTA1 | 0.23 | Spectrin alpha chain, erythrocytic 1 |
| P07738 | BPGM | 0.23 | Bisphosphoglycerate mutase |
| O14558 | HSPB6 | 0.23 | Heat shock protein beta-6 |
| P69905 | HBA1 | 0.23 | Hemoglobin subunit alpha |
| Q9NRN5 | OLFML3 | 0.24 | Olfactomedin-like protein 3 |
| P11277 | SPTB | 0.24 | Spectrin beta chain, erythrocytic |
| P02042 | HBD | 0.25 | Hemoglobin subunit delta |
| P68871 | HBB | 0.25 | Hemoglobin subunit beta |
| P11166 | SLC2A1 | 0.26 | Solute carrier family 2, facilitated glucose transporter member 1 |
| P69891 | HBG1 | 0.26 | Hemoglobin subunit gamma-1 |
| P35670 | ATP7B | 0.28 | Copper-transporting ATPase 2 |
| P27816 | MAP4 | 0.28 | Microtubule-associated protein 4 |
| Q5T619 | ZNF648 | 0.28 | Zinc finger protein 648 |
| O75380 | NDUFS6 | 0.29 | NADH dehydrogenase [ubiquinone] iron-sulfur protein 6, mitochondrial |
| Q92911 | SLC5A5 | 0.30 | Sodium/iodide cotransporter |
| P02511 | CRYAB | 0.31 | Alpha-crystallin B chain |
| Q9BVA1 | TUBB2B | 0.31 | Tubulin beta-2B chain |
| Q96KS0 | EGLN2 | 0.31 | Prolyl hydroxylase EGLN2 |
| P00915 | CA1 | 0.31 | Carbonic anhydrase 1 |
| Q9BXN1 | ASPN | 0.31 | Asporin |
| P22105 | TNXB | 0.31 | Tenascin-X |
| Q13336 | SLC14A1 | 0.32 | Urea transporter 1 |
| P41219 | PRPH | 0.33 | Peripherin |
| P12110 | COL6A2 | 0.33 | Collagen alpha-2(VI) chain |
| P12109 | COL6A1 | 0.34 | Collagen alpha-1(VI) chain |
| Q08495 | DMTN | 0.35 | Dematin |
| A8TX70 | COL6A5 | 0.36 | Collagen alpha-5(VI) chain |
| Q13683 | ITGA7 | 0.38 | Integrin alpha-7 |
| P02100 | HBE1 | 0.38 | Hemoglobin subunit epsilon |
| Q9NYB9 | AB12 | 0.38 | Abl interactor 2 |
| P30613 | PKLR | 0.39 | Pyruvate kinase PKLR |
| Q05707 | COL14A1 | 0.39 | Collagen alpha-1(XIV) chain |
| P22692 | 1GFBP4 | 0.39 | Insulin-like growth factor-binding protein 4 |
| Q9Y6I3 | EPN1 | 0.41 | Epsin-1 |
| P35612 | ADD2 | 0.41 | Beta-adducin |
| Q5XPI4 | RNF123 | 0.42 | E3 ubiquitin-protein ligase RNF123 |
| P0C0L5 | C4B | 0.42 | Complement C4-B |
| Q16527 | CSRP2 | 0.42 | Cysteine and glycine-rich protein 2 |
| P12111 | COL6A3 | 0.43 | Collagen alpha-3(VI) chain |
| Q9UGM5 | FETUB | 0.44 | Fetuin-B |
| Q6P6B1 | ERICH5 | 0.44 | Glutamate-rich protein 5 |
| P11234 | RALB | 0.44 | Ras-related protein Ral-B |
| Q00013 | MPP1 | 0.46 | 55 kDa erythrocyte membrane protein |
| P24844 | MYL9 | 0.47 | Myosin regulatory light polypeptide 9 |
| P00746 | CFD | 0.47 | Complement factor D |
| Q9NZ45 | CISD1 | 0.47 | CDGSH iron-sulfur domain-containing protein 1 |
| P02776 | PF4 | 0.48 | Platelet factor 4 |
| P05387 | RPLP2 | 0.49 | 60S acidic ribosomal protein P2 |
