# Supplementary material for: Novel insights on remnant stomach following Roux-en-Y gastric bypass surgery based on histological evaluation and quantitative proteomics analysis
Source: Sci Rep. 2025 Jul 12;15:25243. doi: 10.1038/s41598-025-10114-x (PMC12255712; doi:10.1038/s41598-025-10114-x)
Supplement: Supplementary file 2 — Supplementary Material 2 [file 41598_2025_10114_MOESM2_ESM.docx]

**Table 2.** List of protein expression in the corpus displaying a fold change greater than 2 (indicating a doubled increase postoperative) or a 2-fold decrease (indicating a 50% decrease perioperative).

| ***Accession Symbol Fold Change Description***  ***Upregulated protein expression*** | | | |
| --- | --- | --- | --- |
| P15085 | CPA1 | 50.28 | Carboxypeptidase A1 |
| P16233 | PNLIP | 39.77 | Pancreatic triacylglycerol lipase |
| P08217 | CELA2A | 19.49 | Chymotrypsin-like elastase family member 2A |
| P08861 | CELA3B | 9.07 | Chymotrypsin-like elastase family member 3B |
| P35556 | FBN2 | 8.89 | Fibrillin-2 |
| P0DUB6 | AMY1A | 8.84 | Alpha-amylase 1A |
| Q8N9E0 | FAM133A | 6.64 | Protein FAM133A |
| Q14031 | COL4A6 | 5.68 | Collagen alpha-6(IV) chain |
| P31350 | RRM2 | 5.15 | Ribonucleoside-diphosphate reductase subunit M2 |
| Q96KB5 | PBK | 4.99 | Lymphokine-activated killer T-cell-originated protein kinase |
| O95239 | KIF4A | 4.98 | Chromosome-associated kinesin KIF4A |
| O14965 | AURKA | 4.89 | Aurora kinase A |
| P35555 | FBN1 | 4.86 | Fibrillin-1 |
| P55001 | MFAP2 | 4.84 | Microfibrillar-associated protein 2 |
| P62861 | FAU | 4.72 | FAU ubiquitin-like and ribosomal protein S30 |
| P52732 | KIF11 | 4.63 | Kinesin-like protein KIF11 |
| Q8IYB3 | SRRM1 | 4.34 | Serine/arginine repetitive matrix protein 1 |
| Q8N2A8 | PLD6 | 4.30 | Mitochondrial cardiolipin hydrolase |
| Q15645 | TRIP13 | 4.27 | Pachytene checkpoint protein 2 homolog |
| Q15021 | NCAPD2 | 4.22 | Condensin complex subunit 1 |
| P18621 | RPL17 | 4.19 | 60S ribosomal protein L17 |
| P61927 | RPL37 | 4.17 | 60S ribosomal protein L37 |
| P42766 | RPL35 | 4.08 | 60S ribosomal protein L35 |
| Q08170 | SRSF4 | 4.03 | Serine/arginine-rich splicing factor 4 |
| Q86VI3 | IQGAP3 | 3.98 | Ras GTPase-activating-like protein IQGAP3 |
| P46776 | RPL27A | 3.95 | 60S ribosomal protein L27a |
| Q02388 | COL7A1 | 3.95 | Collagen alpha-1(VII) chain |
| P15086 | CPB1 | 3.95 | Carboxypeptidase B |
| P02786 | TFRC | 3.87 | Transferrin receptor protein 1 |
| Q9BXW9 | FANCD2 | 3.85 | Fanconi anemia group D2 protein |
| Q9BW19 | KIFC1 | 3.83 | Kinesin-like protein KIFC1 |
| O75394 | MRPL33 | 3.78 | 39S ribosomal protein L33, mitochondrial |
| Q02539 | H1-1 | 3.74 | Histone H1.1 |
| Q8N2S1 | LTBP4 | 3.74 | Latent-transforming growth factor beta-binding protein 4 |
| Q15287 | RNPS1 | 3.67 | RNA-binding protein with serine-rich domain 1 |
| P15085 | DDC | 3.66 | Aromatic-L-amino-acid decarboxylase |
| P50748 | KNTC1 | 3.64 | Kinetochore-associated protein 1 |
| P26373 | RPL13 | 3.59 | 60S ribosomal protein L13 |
| P16233 | RPL36AL | 3.56 | 60S ribosomal protein L36a-like |
| Q9BPX3 | NCAPG | 3.53 | Condensin complex subunit 3 |
| Q9NS87 | KIF15 | 3.52 | Kinesin-like protein KIF15 |
| P02462 | COL4A1 | 3.49 | Collagen alpha-1(IV) chain |
| P08217 | NOLC1 | 3.48 | Nucleolar and coiled-body phosphoprotein 1 |
| P08861 | RPL21 | 3.39 | 60S ribosomal protein L21 |
| Q8N9E1 | MCM6 | 3.32 | DNA replication licensing factor MCM6 |
| P35556 | RPL22L1 | 3.31 | 60S ribosomal protein L22-like 1 |
| P06493 | CDK1 | 3.30 | Cyclin-dependent kinase 1 |
| Q13257 | MAD2L1 | 3.28 | Mitotic spindle assembly checkpoint protein MAD2A |
| P0DUB7 | TOP2A | 3.26 | DNA topoisomerase 2-alpha |
| P25205 | MCM3 | 3.26 | DNA replication licensing factor MCM3 |
| Q9Y6C2 | EMILIN1 | 3.24 | EMILIN-1 |
| P46779 | RPL28 | 3.18 | 60S ribosomal protein L28 |
| P47914 | RPL29 | 3.17 | 60S ribosomal protein L29 |
| Q2NKX8 | ERCC6L | 3.15 | DNA excision repair protein ERCC-6-like |
| Q6UXI9 | NPNT | 3.13 | Nephronectin |
| P49736 | MCM2 | 3.08 | DNA replication licensing factor MCM2 |
| O95229 | ZWINT | 3.05 | ZW10 interactor |
| P33991 | MCM4 | 3.03 | DNA replication licensing factor MCM4 |
| Q14766 | LTBP1 | 3.02 | Latent-transforming growth factor beta-binding protein 1 |
| Q9UH17 | APOBEC3B | 3.02 | DNA dC->dU-editing enzyme APOBEC-3B |
| P16401 | H1-5 | 3.02 | Histone H1.5 |
| P52292 | KPNA2 | 3.01 | Importin subunit alpha-1 |
| Q14680 | MELK | 3.00 | Maternal embryonic leucine zipper kinase |
| Q13753 | LAMC2 | 2.98 | Laminin subunit gamma-2 |
| Q16787 | LAMA3 | 2.90 | Laminin subunit alpha-3 |
| P08572 | COL4A2 | 2.89 | Collagen alpha-2(IV) chain |
| O7568 | SURF6 | 2.86 | Surfeit locus protein 6 |
| P62910 | RPL32 | 2.83 | 60S ribosomal protein L32 |
| O14757 | CHEK1 | 2.82 | Serine/threonine-protein kinase Chk1 |
| P61254 | RPL26 | 2.78 | 60S ribosomal protein L26 |
| Q8WVK2 | SNRNP27 | 2.77 | U4/U6.U5 small nuclear ribonucleoprotein 27 kDa protein |
| P63173 | RPL38 | 2.76 | 60S ribosomal protein L38 |
| O95235 | KIF20A | 2.76 | Kinesin-like protein KIF20A |
| O95395 | GCNT3 | 2.76 | Beta-1,3-galactosyl-O-glycosyl-glycoprotein beta-1,6-N-acetylglucosaminyltransferase 3 |
| Q9UKJ3 | GPATCH8 | 2.73 | G patch domain-containing protein 8 |
| Q13751 | LAMB3 | 2.71 | Laminin subunit beta-3 |
| P61513 | RPL37A | 2.69 | 60S ribosomal protein L37a |
| Q9Y3Y2 | CHTOP | 2.66 | Chromatin target of PRMT1 protein |
| O95347 | SMC2 | 2.65 | Structural maintenance of chromosomes protein 2 |
| O95967 | EFEMP2 | 2.62 | EGF-containing fibulin-like extracellular matrix protein 2 |
| O95218 | ZRANB2 | 2.61 | Zinc finger Ran-binding domain-containing protein 2 O |
| P83731 | RPL24 | 2.56 | 60S ribosomal protein L24 |
| Q14541 | HNF4G | 2.53 | Hepatocyte nuclear factor 4-gamma |
| P60866 | RPS20 | 2.51 | 40S ribosomal protein S20 |
| Q9UKE5 | TNIK | 2.51 | TRAF2 and NCK-interacting protein kinase |
| P46087 | NOP2 | 2.50 | Probable 28S rRNA (cytosine(4447)-C(5))-methyltransferase |
| Q14142 | TRIM14 | 2.49 | Tripartite motif-containing protein 14 |
| Q6ZMB0 | B3GNT6 | 2.49 | Acetylgalactosaminyl-O-glycosyl-glycoprotein beta-1,3-N-acetylglucosaminyltransferase |
| P37268 | FDFT1 | 2.47 | Squalene synthase |
| P27635 | RPL10 | 2.47 | 60S ribosomal protein L10 |
| P62266 | RPS23 | 2.45 | 40S ribosomal protein S23 |
| O75607 | NPM3 | 2.45 | Nucleoplasmin-3 |
| P33993 | MCM7 | 2.43 | DNA replication licensing factor MCM7 |
| Q9NTJ3 | SMC4 | 2.42 | Structural maintenance of chromosomes protein 4 |
| P33992 | MCM5 | 2.42 | DNA replication licensing factor MCM5 |
| Q9NR30 | DDX21 | 2.39 | Nucleolar RNA helicase 2 |
| Q6UX53 | METTL7B | 2.37 | Thiol S-methyltransferase METTL7B |
| O15347 | HMGB3 | 2.35 | High mobility group protein B3 |
| Q9H900 | ZWILCH | 2.34 | Protein zwilch homolog |
| P49207 | RPL34 | 2.33 | 60S ribosomal protein L34 |
| Q9H3R2 | MUC13 | 2.33 | Mucin-13 |
| Q9NX58 | LYAR | 2.31 | Cell growth-regulating nucleolar protein |
| P42696 | RBM34 | 2.31 | RNA-binding protein 34 |
| Q9H9Y2 | RPF1 | 2.30 | Ribosome production factor 1 |
| Q9NWH9 | SLTM | 2.29 | SAFB-like transcription modulator |
| Q8WVX9 | FAR1 | 2.28 | Fatty acyl-CoA reductase 1 |
| Q9BW71 | HIRIP3 | 2.27 | HIRA-interacting protein 3 |
| Q9H8V3 | ECT2 | 2.26 | Protein ECT2 |
| P62753 | RPS6 | 2.26 | 40S ribosomal protein S6 |
| P62917 | RPL8 | 2.25 | 60S ribosomal protein L8 |
| Q8IYS1 | PM20D2 | 2.24 | Xaa-Arg dipeptidase |
| Q8TE96 | DQX1 | 2.24 | ATP-dependent RNA helicase DQX1 |
| Q16695 | H3-4 | 2.23 | Histone H3.1t |
| Q14534 | SQLE | 2.22 | Squalene monooxygenase |
| Q96E22 | NUS1 | 2.22 | Dehydrodolichyl diphosphate synthase complex subunit NUS1 |
| P35269 | GTF2F1 | 2.19 | General transcription factor IIF subunit 1 |
| P09884 | POLA1 | 2.19 | DNA polymerase alpha catalytic subunit |
| Q9NSI6 | BRWD1 | 2.19 | Bromodomain and WD repeat-containing protein 1 |
| O00148 | DDX39A | 2.18 | ATP-dependent RNA helicase DDX39A |
| P78545 | ELF3 | 2.17 | ETS-related transcription factor Elf-3 |
| Q9UGU0 | TCF20 | 2.17 | Transcription factor 20 |
| P40429 | RPL13A | 2.16 | 60S ribosomal protein L13a |
| Q9ULI0 | ATAD2B | 2.16 | ATPase family AAA domain-containing protein 2B |
| Q13428 | TCOF1 | 2.16 | Treacle protein |
| O15427 | SLC16A3 | 2.16 | Monocarboxylate transporter 4 |
| P49327 | FASN | 2.15 | Fatty acid synthase |
| Q8N567 | ZCCHC9 | 2.15 | Zinc finger CCHC domain-containing protein 9 |
| O75400 | PRPF40A | 2.14 | Pre-mRNA-processing factor 40 homolog A |
| Q9Y221 | NIP7 | 2.14 | 60S ribosome subunit biogenesis protein NIP7 homolog |
| O43818 | RRP9 | 2.13 | U3 small nucleolar RNA-interacting protein 2 |
| Q8WTV0 | SCARB1 | 2.13 | Scavenger receptor class B member 1 |
| Q9Y3B9 | RRP15 | 2.12 | RRP15-like protein |
| P39019 | RPS19 | 2.11 | 40S ribosomal protein S19 |
| Q53FV1 | ORMDL2 | 2.10 | ORM1-like protein 2 |
| Q9NNW5 | WDR6 | 2.10 | WD repeat-containing protein 6 |
| Q93088 | BHMT | 2.09 | Betaine--homocysteine S-methyltransferase 1 |
| Q12805 | EFEMP1 | 2.08 | EGF-containing fibulin-like extracellular matrix protein 1 |
| O43291 | SPINT2 | 2.08 | Kunitz-type protease inhibitor 2 |
| Q96B70 | LENG9 | 2.07 | Leukocyte receptor cluster member 9 |
| P36578 | RPL4 | 2.07 | 60S ribosomal protein L4 |
| P62851 | RPS25 | 2.07 | 40S ribosomal protein S25 |
| O00541 | PES1 | 2.05 | Pescadillo homolog |
| Q9NRL2 | BAZ1A | 2.04 | Bromodomain adjacent to zinc finger domain protein 1A |
| P06727 | APOA4 | 2.03 | Apolipoprotein A-IV |
| P62899 | RPL31 | 2.02 | 60S ribosomal protein L31 |
| Q9BQL6 | FERMT1 | 2.02 | Fermitin family homolog 1 |
| O43688 | PLPP2 | 2.02 | Phospholipid phosphatase 2 |
| O75762 | TRPA1 | 2.01 | Transient receptor potential cation channel subfamily A member 1 |
| P08708 | RPS17 | 2.00 | 40S ribosomal protein S17 |
| ***Accession Symbol Fold Change Description***  ***Downregulated protein expression*** | | | |
| P15104 | GLUL | 0.21 | Glutamine synthetase |
| P0DJD7 | PGA4 | 0.22 | Pepsin A-4 |
| P05413 | FABP3 | 0.25 | Fatty acid-binding protein, heart |
| Q06520 | SULT2A1 | 0.25 | Sulfotransferase 2A1 |
| O14561 | NDUFAB1 | 0.28 | Acyl carrier protein, mitochondrial |
| P55809 | OXCT1 | 0.29 | Succinyl-CoA:3-ketoacid coenzyme A transferase 1, mitochondrial |
| O75380 | NDUFS6 | 0.29 | NADH dehydrogenase [ubiquinone] iron-sulfur protein 6, mitochondrial |
| Q8N3J6 | CADM2 | 0.31 | Cell adhesion molecule 2 |
| P02794 | FTH1 | 0.32 | Ferritin heavy chain |
| P02792 | FTL | 0.32 | Ferritin light chain |
| Q9UHK6 | AMACR | 0.32 | Alpha-methylacyl-CoA racemase |
| Q7Z3F1 | GPR155 | 0.35 | Integral membrane protein GPR155 |
| Q9GZV4 | EIF5A2 | 0.36 | Eukaryotic translation initiation factor 5A-2 |
| Q16798 | ME3 | 0.36 | NADP-dependent malic enzyme, mitochondrial |
| P24752 | ACAT1 | 0.37 | Acetyl-CoA acetyltransferase, mitochondrial |
| O43181 | NDUFS4 | 0.38 | NADH dehydrogenase [ubiquinone] iron-sulfur protein 4, mitochondrial |
| P17540 | CKMT2 | 0.38 | Creatine kinase S-type, mitochondrial |
| Q86WU2 | LDHD | 0.38 | Probable D-lactate dehydrogenase, mitochondrial |
| P12277 | CKB | 0.39 | Creatine kinase B-type |
| O00217 | NDUFS8 | 0.39 | NADH dehydrogenase [ubiquinone] iron-sulfur protein 8, mitochondrial |
| P10606 | COX5B | 0.39 | Cytochrome c oxidase subunit 5B, mitochondrial |
| Q99798 | ACO2 | 0.39 | Aconitate hydratase, mitochondrial |
| P01040 | CSTA | 0.40 | Cystatin-A |
| P06732 | CKM | 0.41 | Creatine kinase M-type |
| Q9Y2E5 | MAN2B2 | 0.41 | Epididymis-specific alpha-mannosidase |
| O95298 | NDUFC2 | 0.41 | NADH dehydrogenase [ubiquinone] 1 subunit C2 |
| P27352 | CBLIF | 0.42 | Cobalamin binding intrinsic factor |
| Q96RQ3 | MCCC1 | 0.42 | Methylcrotonoyl-CoA carboxylase subunit alpha, mitochondrial |
| Q9NZ45 | CISD1 | 0.42 | CDGSH iron-sulfur domain-containing protein 1 |
| P48735 | IDH2 | 0.43 | Isocitrate dehydrogenase [NADP], mitochondrial |
| P20648 | ATP4A | 0.43 | Potassium-transporting ATPase alpha chain 1 |
| P12694 | BCKDHA | 0.43 | 2-oxoisovalerate dehydrogenase subunit alpha, mitochondrial |
| O95299 | NDUFA10 | 0.43 | NADH dehydrogenase [ubiquinone] 1 alpha subcomplex subunit 10, mitochondrial |
| P15090 | FABP4 | 0.44 | Fatty acid-binding protein, adipocyte |
| Q16134 | ETFDH | 0.44 | Electron transfer flavoprotein-ubiquinone oxidoreductase, mitochondrial |
| O95182 | NDUFA7 | 0.44 | NADH dehydrogenase [ubiquinone] 1 alpha subcomplex subunit 7 |
| Q8N0X4 | CLYBL | 0.45 | Citramalyl-CoA lyase, mitochondrial |
| O43678 | NDUFA2 | 0.45 | NADH dehydrogenase [ubiquinone] 1 alpha subcomplex subunit 2 |
| Q86XT4 | TRIM50 | 0.45 | E3 ubiquitin-protein ligase TRIM50 |
| P11182 | DBT | 0.46 | Lipoamide acyltransferase component of branched-chain alpha-keto acid dehydrogenase complex, mitochondrial |
| Q9H061 | TMEM126A | 0.46 | Transmembrane protein 126A |
| P24298 | GPT | 0.46 | Alanine aminotransferase 1 |
| O95167 | NDUFA3 | 0.46 | NADH dehydrogenase [ubiquinone] 1 alpha subcomplex subunit 3 |
| Q7LBE3 | SLC26A9 | 0.47 | Solute carrier family 26 member 9 |
| Q9NWU1 | OXSM | 0.47 | 3-oxoacyl-[acyl-carrier-protein] synthase, mitochondrial |
| P48052 | CPA2 | 0.47 | Carboxypeptidase A2 |
| P03897 | MT-ND3 | 0.47 | NADH-ubiquinone oxidoreductase chain 3 |
| Q86YB7 | ECHDC2 | 0.48 | Enoyl-CoA hydratase domain-containing protein 2, mitochondrial |
| Q9P0J0 | NDUFA13 | 0.48 | NADH dehydrogenase [ubiquinone] 1 alpha subcomplex subunit 13 |
| O75438 | NDUFB1 | 0.48 | NADH dehydrogenase [ubiquinone] 1 beta subcomplex subunit 1 |
| Q6P461 | ACSM6 | 0.49 | Acyl-coenzyme A synthetase ACSM6, mitochondrial |
| O95822 | MLYCD | 0.49 | Malonyl-CoA decarboxylase, mitochondrial |
| O75306 | NDUFS2 | 0.49 | NADH dehydrogenase [ubiquinone] iron-sulfur protein 2, mitochondrial |
| Q15111 | PLCL1 | 0.50 | Inactive phospholipase C-like protein 1 |
| Q8N442 | GUF1 | 0.50 | Translation factor GUF1, mitochondrial |
