# Supplementary material for: Novel insights on remnant stomach following Roux-en-Y gastric bypass surgery based on histological evaluation and quantitative proteomics analysis
Source: Sci Rep. 2025 Jul 12;15:25243. doi: 10.1038/s41598-025-10114-x (PMC12255712; doi:10.1038/s41598-025-10114-x)
Supplement: Supplementary file 3 — Supplementary Material 3 [file 41598_2025_10114_MOESM3_ESM.docx]

**Table 3.** List of protein expression in the antrum displaying a fold change greater than 2 (indicating a doubled increase postoperative) or a 2-fold decrease (indicating a 50% decrease perioperative).

| ***Accession Symbol Fold Change Description***  ***Upregulated protein expression*** | | | |
| --- | --- | --- | --- |
| P16233 | PNLIP | 39.94 | Pancreatic triacylglycerol lipase |
| P35556 | FBN2 | 10.07 | Fibrillin-2 |
| P55259 | GP2 | 9.01 | Pancreatic secretory granule membrane major glycoprotein GP2 |
| P31350 | RRM2 | 7.08 | Ribonucleoside-diphosphate reductase subunit M2 |
| O15392 | BIRC5 | 7.01 | Baculoviral IAP repeat-containing protein 5 |
| Q96T88 | UHRF1 | 6.95 | E3 ubiquitin-protein ligase UHRF1 |
| Q9BXW9 | FANCD2 | 6.54 | Fanconi anemia group D2 protein |
| Q8N4L8 | CCDC24 | 6.50 | Coiled-coil domain-containing protein 24 |
| Q02388 | COL7A1 | 6.46 | Collagen alpha-1(VII) chain |
| O95229 | ZWINT | 6.22 | ZW10 interactor |
| Q96KB5 | PBK | 6.16 | Lymphokine-activated killer T-cell-originated protein kinase |
| Q9BPX3 | NCAPG | 5.86 | Condensin complex subunit 3 |
| Q15021 | NCAPD2 | 5.84 | Condensin complex subunit 1 |
| Q9NRZ9 | HELLS | 5.79 | Lymphoid-specific helicase |
| O95239 | KIF4A | 5.71 | Chromosome-associated kinesin KIF4A |
| Q86VI3 | IQGAP3 | 5.58 | Ras GTPase-activating-like protein IQGAP3 |
| P29120 | PCSK1 | 5.55 | Neuroendocrine convertase 1 |
| Q9H900 | ZWILCH | 5.48 | Protein zwilch homolog |
| O00762 | UBE2C | 5.38 | Ubiquitin-conjugating enzyme E2 C |
| Q14680 | MELK | 5.33 | Maternal embryonic leucine zipper kinase |
| Q14566 | MCM6 | 5.26 | DNA replication licensing factor MCM6 |
| P25205 | MCM3 | 5.03 | DNA replication licensing factor MCM3 |
| Q9H7Z3 | NRDE2 | 4.94 | Nuclear exosome regulator NRDE2 |
| Q15645 | TRIP13 | 4.88 | Pachytene checkpoint protein 2 homolog |
| P36952 | SERPINB5 | 4.80 | Serpin B5 |
| Q6PL18 | ATAD2 | 4.70 | ATPase family AAA domain-containing protein 2 |
| Q9UNS1 | TIMELESS | 4.68 | Protein timeless homolog |
| P49736 | MCM2 | 4.59 | DNA replication licensing factor MCM2 |
| P33991 | MCM4 | 4.54 | DNA replication licensing factor MCM4 |
| P49454 | CENPF | 4.52 | Centromere protein F |
| P35555 | FBN1 | 4.45 | Fibrillin-1 |
| Q08170 | SRSF4 | 4.37 | Serine/arginine-rich splicing factor 4 |
| O75717 | WDHD1 | 4.37 | WD repeat and HMG-box DNA-binding protein 1 |
| O95067 | CCNB2 | 4.33 | G2/mitotic-specific cyclin-B2 |
| Q14031 | COL4A6 | 4.32 | Collagen alpha-6(IV) chain |
| Q9NSV4 | DIAPH3 | 4.15 | Protein diaphanous homolog 3 |
| Q13257 | MAD2L1 | 4.11 | Mitotic spindle assembly checkpoint protein MAD2A |
| P16870 | CPE | 4.05 | Carboxypeptidase E |
| P02786 | TFRC | 4.00 | Transferrin receptor protein 1 |
| Q9NS87 | KIF15 | 3.96 | Kinesin-like protein KIF15 |
| P27930 | IL1R2 | 3.91 | Interleukin-1 receptor type 2 |
| Q9BSE5 | AGMAT | 3.83 | Agmatinase, mitochondrial |
| P11388 | TOP2A | 3.80 | DNA topoisomerase 2-alpha |
| P35269 | GTF2F1 | 3.73 | General transcription factor IIF subunit 1 |
| Q8TA86 | RP9 | 3.72 | Retinitis pigmentosa 9 protein |
| Q9NYT0 | PLEK2 | 3.70 | Pleckstrin-2 |
| Q13309 | SKP2 | 3.67 | S-phase kinase-associated protein 2 |
| O95833 | CLIC3 | 3.67 | Chloride intracellular channel protein 3 |
| P06493 | CDK1 | 3.65 | Cyclin-dependent kinase 1 |
| Q9UGY1 | NOL12 | 3.63 | Nucleolar protein 12 |
| O75144 | ICOSLG | 3.61 | ICOS ligand |
| O43543 | XRCC2 | 3.60 | DNA repair protein XRCC2 |
| Q8N2S1 | LTBP4 | 3.60 | Latent-transforming growth factor beta-binding protein 4 |
| P20337 | RAB3B | 3.57 | Ras-related protein Rab-3B |
| P52292 | KPNA2 | 3.56 | Importin subunit alpha-1 |
| P23921 | RRM1 | 3.56 | Ribonucleoside-diphosphate reductase large subunit |
| Q9H3R5 | CENPH | 3.53 | Centromere protein H |
| Q6P5R6 | RPL22L1 | 3.51 | 60S ribosomal protein L22-like 1 |
| O14578 | CIT | 3.50 | Citron Rho-interacting kinase |
| Q8WVK2 | SNRNP27 | 3.48 | U4/U6.U5 small nuclear ribonucleoprotein 27 kDa protein |
| Q9NRS4 | TMPRSS4 | 3.43 | Transmembrane protease serine 4 |
| Q13427 | PPIG | 3.43 | Peptidyl-prolyl cis-trans isomerase G |
| Q13751 | LAMB3 | 3.41 | Laminin subunit beta-3 |
| Q8N398 | VWA5B2 | 3.41 | von Willebrand factor A domain-containing protein 5B2 |
| Q16787 | LAMA3 | 3.38 | Laminin subunit alpha-3 |
| Q01581 | HMGCS1 | 3.38 | Hydroxymethylglutaryl-CoA synthase, cytoplasmic |
| P33992 | MCM5 | 3.29 | DNA replication licensing factor MCM5 |
| Q8WXD2 | SCG3 | 3.28 | Secretogranin-3 |
| O95347 | SMC2 | 3.24 | Structural maintenance of chromosomes protein 2 |
| Q9NTJ3 | SMC4 | 3.22 | Structural maintenance of chromosomes protein 4 |
| P47914 | RPL29 | 3.21 | 60S ribosomal protein L29 |
| P28715 | ERCC5 | 3.20 | DNA excision repair protein ERCC-5 |
| Q13753 | LAMC2 | 3.20 | Laminin subunit gamma-2 |
| Q14978 | NOLC1 | 3.18 | Nucleolar and coiled-body phosphoprotein 1 |
| Q7Z624 | CAMKMT | 3.13 | Calmodulin-lysine N-methyltransferase |
| P42695 | NCAPD3 | 3.13 | Condensin-2 complex subunit D3 |
| O14965 | AURKA | 3.11 | Aurora kinase A |
| P09884 | POLA1 | 3.11 | DNA polymerase alpha catalytic subunit |
| P37268 | FDFT1 | 3.10 | Squalene synthase |
| O75394 | MRPL33 | 3.08 | 39S ribosomal protein L33, mitochondrial |
| P61927 | RPL37 | 3.08 | 60S ribosomal protein L37 |
| O60870 | KIN | 3.06 | DNA/RNA-binding protein KIN17 |
| Q8TE96 | DQX1 | 3.02 | ATP-dependent RNA helicase DQX1 |
| Q9H857 | NT5DC2 | 3.02 | 5'-nucleotidase domain-containing protein 2 |
| Q9UH17 | APOBEC3B | 3.01 | DNA dC->dU-editing enzyme APOBEC-3B |
| O43688 | PLPP2 | 3.00 | Phospholipid phosphatase 2 |
| P33993 | MCM7 | 3.00 | DNA replication licensing factor MCM7 |
| Q15287 | RNPS1 | 3.00 | RNA-binding protein with serine-rich domain 1 |
| P16401 | H1-5 | 2.99 | Histone H1.5 |
| Q6IPU0 | CENPP | 2.97 | Centromere protein P |
| Q8IW92 | GLB1L2 | 2.97 | Beta-galactosidase-1-like protein 2 |
| P18621 | RPL17 | 2.96 | 60S ribosomal protein L17 |
| O76038 | SCGN | 2.95 | Secretagogin |
| Q9BW71 | HIRIP3 | 2.93 | HIRA-interacting protein 3 |
| Q9Y6C2 | EMILIN1 | 2.91 | EMILIN-1 |
| P16422 | EPCAM | 2.84 | Epithelial cell adhesion molecule |
| P46087 | NOP2 | 2.83 | Probable 28S rRNA (cytosine(4447)-C(5))-methyltransferase |
| P42766 | RPL35 | 2.82 | 60S ribosomal protein L35 |
| Q14766 | LTBP1 | 2.79 | Latent-transforming growth factor beta-binding protein 1 |
| O43291 | SPINT2 | 2.78 | Kunitz-type protease inhibitor 2 |
| P46778 | RPL21 | 2.77 | 60S ribosomal protein L21 |
| Q14691 | GINS1 | 2.74 | DNA replication complex GINS protein PSF1 |
| Q9NR30 | DDX21 | 2.74 | Nucleolar RNA helicase 2 |
| Q8IYB3 | SRRM1 | 2.74 | Serine/arginine repetitive matrix protein 1 |
| O60287 | URB1 | 2.72 | Nucleolar pre-ribosomal-associated protein 1 |
| Q96B70 | LENG9 | 2.72 | Leukocyte receptor cluster member 9 |
| Q96E22 | NUS1 | 2.68 | Dehydrodolichyl diphosphate synthase complex subunit NUS1 |
| Q8TCT7 | SPPL2B | 2.68 | Signal peptide peptidase-like 2B |
| Q9ULI0 | ATAD2B | 2.66 | ATPase family AAA domain-containing protein 2B |
| Q96EP1 | CHFR | 2.64 | E3 ubiquitin-protein ligase CHFR |
| Q14534 | SQLE | 2.64 | Squalene monooxygenase |
| Q8NCN4 | RNF169 | 2.62 | E3 ubiquitin-protein ligase RNF169 |
| Q5T280 | SPOUT1 | 2.61 | Putative methyltransferase C9orf114 |
| O95478 | NSA2 | 2.58 | Ribosome biogenesis protein NSA2 homolog |
| Q8TEX9 | IPO4 | 2.57 | Importin-4 |
| Q6UWR7 | ENPP6 | 2.57 | Glycerophosphocholine cholinephosphodiesterase ENPP6 |
| Q15389 | ANGPT1 | 2.57 | Angiopoietin-1 |
| P62861 | FAU | 2.55 | FAU ubiquitin-like and ribosomal protein S30 |
| O14929 | HAT1 | 2.55 | Histone acetyltransferase type B catalytic subunit |
| P49327 | FASN | 2.54 | Fatty acid synthase |
| Q8TE68 | EPS8L1 | 2.53 | Epidermal growth factor receptor kinase substrate 8-like protein 1 |
| P63173 | RPL38 | 2.53 | 60S ribosomal protein L38 |
| Q16850 | CYP51A1 | 2.52 | Lanosterol 14-alpha demethylase |
| Q15800 | MSMO1 | 2.52 | Methylsterol monooxygenase 1 |
| O14646 | CHD1 | 2.46 | Chromodomain-helicase-DNA-binding protein 1 |
| P62910 | RPL32 | 2.43 | 60S ribosomal protein L32 |
| Q16763 | UBE2S | 2.42 | Ubiquitin-conjugating enzyme E2 S |
| Q9NR33 | POLE4 | 2.41 | DNA polymerase epsilon subunit 4 |
| O15554 | KCNN4 | 2.40 | Intermediate conductance calcium-activated potassium channel protein 4 |
| Q14692 | BMS1 | 2.40 | Ribosome biogenesis protein BMS1 homolog |
| P49642 | PRIM1 | 2.39 | DNA primase small subunit |
| Q9BU76 | MMTAG2 | 2.38 | Multiple myeloma tumor-associated protein 2 |
| P46776 | RPL27A | 2.38 | 60S ribosomal protein L27a |
| Q8NBT0 | POC1A | 2.37 | POC1 centriolar protein homolog A |
| P48436 | SOX9 | 2.37 | Transcription factor SOX-9 |
| P26373 | RPL13 | 2.37 | 60S ribosomal protein L13 |
| O15347 | HMGB3 | 2.36 | High mobility group protein B3 |
| Q9BQG0 | MYBBP1A | 2.35 | Myb-binding protein 1A |
| P41235 | HNF4A | 2.34 | Hepatocyte nuclear factor 4-alpha |
| Q9NRX1 | PNO1 | 2.33 | RNA-binding protein PNO1 |
| Q9H6R4 | NOL6 | 2.33 | Nucleolar protein 6 |
| Q9NV31 | IMP3 | 2.33 | U3 small nucleolar ribonucleoprotein protein IMP3 |
| P39748 | FEN1 | 2.30 | Flap endonuclease 1 |
| Q8NAF0 | ZNF579 | 2.29 | Zinc finger protein 579 |
| P62314 | SNRPD1 | 2.29 | Small nuclear ribonucleoprotein Sm D1 |
| Q9BVI4 | NOC4L | 2.28 | Nucleolar complex protein 4 homolog |
| Q6FIF0 | ZFAND6 | 2.28 | AN1-type zinc finger protein 6 |
| P26358 | DNMT1 | 2.28 | DNA (cytosine-5)-methyltransferase 1 |
| P28340 | POLD1 | 2.27 | DNA polymerase delta catalytic subunit |
| Q9UJK0 | TSR3 | 2.27 | 18S rRNA aminocarboxypropyltransferase |
| P61024 | CKS1B | 2.27 | Cyclin-dependent kinases regulatory subunit 1 |
| P78504 | JAG1 | 2.27 | Protein jagged-1 |
| P42696 | RBM34 | 2.26 | RNA-binding protein 34 |
| O00541 | PES1 | 2.26 | Pescadillo homolog |
| O43818 | RRP9 | 2.26 | U3 small nucleolar RNA-interacting protein 2 |
| O75366 | AVIL | 2.26 | Advillin |
| O43734 | TRAF3IP2 | 2.24 | E3 ubiquitin ligase TRAF3IP2 |
| O14975 | SLC27A2 | 2.24 | Long-chain fatty acid transport protein 2 |
| P17301 | ITGA2 | 2.24 | Integrin alpha-2 |
| Q93088 | BHMT | 2.20 | Betaine--homocysteine S-methyltransferase 1 |
| Q09472 | EP300 | 2.20 | Histone acetyltransferase p300 |
| Q8IX07 | FPM1 | 2.20 | Zinc finger protein ZFPM1 |
| Q6UX53 | METTL7B | 2.20 | Thiol S-methyltransferase METTL7B |
| P39019 | RPS19 | 2.20 | 40S ribosomal protein S19 |
| Q5TEC6 | H3-7 | 2.20 | Histone H3-7 |
| Q6DKI1 | RPL7L1 | 2.19 | 60S ribosomal protein L7-like 1 |
| Q8WVX9 | FAR1 | 2.19 | Fatty acyl-CoA reductase 1 |
| P61254 | RPL26 | 2.19 | 60S ribosomal protein L26 |
| O60832 | DKC1 | 2.18 | H/ACA ribonucleoprotein complex subunit DKC1 |
| P00374 | DHFR | 2.18 | Dihydrofolate reductase |
| P12268 | IMPDH2 | 2.18 | Inosine-5'-monophosphate dehydrogenase 2 |
| Q92922 | SMARCC1 | 2.17 | SWI/SNF complex subunit SMARCC1 |
| P60866 | RPS20 | 2.17 | 40S ribosomal protein S20 |
| Q9Y6R1 | SLC4A4 | 2.16 | Electrogenic sodium bicarbonate cotransporter 1 |
| Q6ZMB0 | B3GNT6 | 2.16 | Acetylgalactosaminyl-O-glycosyl-glycoprotein beta-1,3-N-acetylglucosaminyltransferase |
| Q9NUK0 | MBNL3 | 2.15 | Muscleblind-like protein 3 |
| Q9Y289 | SLC5A6 | 2.14 | Sodium-dependent multivitamin transporter |
| P35250 | RFC2 | 2.13 | Replication factor C subunit 2 |
| Q13243 | SRSF5 | 2.13 | Serine/arginine-rich splicing factor 5 |
| P46779 | RPL28 | 2.13 | 60S ribosomal protein L28 |
| Q9H9Y2 | RPF1 | 2.12 | Ribosome production factor 1 |
| P29317 | EPHA2 | 2.12 | Ephrin type-A receptor 2 |
| Q07955 | SRSF1 | 2.12 | Serine/arginine-rich splicing factor 1 |
| Q13895 | BYSL | 2.12 | Bystin |
| P52701 | MSH6 | 2.11 | DNA mismatch repair protein Msh6 |
| P27635 | RPL10 | 2.11 | 60S ribosomal protein L10 |
| O95602 | POLR1A | 2.11 | DNA-directed RNA polymerase I subunit RPA1 |
| O00116 | AGPS | 2.10 | Alkyldihydroxyacetonephosphate synthase, peroxisomal |
| Q9H6Y2 | WDR55 | 2.10 | WD repeat-containing protein 55 |
| Q14676 | MDC1 | 2.10 | Mediator of DNA damage checkpoint protein 1 |
| Q9H173 | SIL1 | 2.10 | Nucleotide exchange factor SIL1 |
| P20585 | MSH3 | 2.10 | DNA mismatch repair protein Msh3 |
| P13984 | GTF2F2 | 2.10 | General transcription factor IIF subunit 2 |
| O75400 | PRPF40A | 2.09 | Pre-mRNA-processing factor 40 homolog A |
| Q86V21 | AACS | 2.08 | Acetoacetyl-CoA synthetase |
| Q8N954 | GPATCH11 | 2.08 | G patch domain-containing protein 11 |
| Q96AY3 | FKBP10 | 2.08 | Peptidyl-prolyl cis-trans isomerase FKBP10 |
| D6REC4 | CFAP99 | 2.08 | Cilia- and flagella-associated protein 99 |
| Q15269 | PWP2 | 2.07 | Periodic tryptophan protein 2 homolog |
| Q8TCG1 | CIP2A | 2.07 | Protein CIP2A |
| Q15649 | ZNHIT3 | 2.06 | Zinc finger HIT domain-containing protein 3 |
| Q9H6R0 | DHX33 | 2.06 | ATP-dependent RNA helicase DHX33 |
| Q9UK23 | NAGPA | 2.04 | N-acetylglucosamine-1-phosphodiester alpha-N-acetylglucosaminidase |
| Q03701 | CEBPZ | 2.04 | CCAAT/enhancer-binding protein zeta |
| P62266 | RPS23 | 2.03 | 40S ribosomal protein S23 |
| Q8WVV4 | POF1B | 2.03 | Protein POF1B |
| Q10472 | GALNT1 | 2.03 | Polypeptide N-acetylgalactosaminyltransferase 1 |
| Q15397 | PUM3 | 2.03 | Pumilio homolog 3 |
| Q8TDN6 | BRIX1 | 2.02 | Ribosome biogenesis protein BRX1 homolog |
| P40937 | RFC5 | 2.02 | Replication factor C subunit 5 |
| Q9BSC4 | NOL10 | 2.02 | Nucleolar protein 10 |
| Q5JTH9 | RRP12 | 2.01 | RRP12-like protein |
| ***Accession Symbol Fold Change Description***  ***Downregulated protein expression*** | | | |
| P49888 | SULT1E1 | 0.12 | Sulfotransferase 1E1 |
| P40394 | ADH7 | 0.14 | All-trans-retinol dehydrogenase [NAD(+)] ADH7 |
| P15104 | GLUL | 0.16 | Glutamine synthetase |
| Q9NRD8 | DUOX2 | 0.17 | Dual oxidase 2 |
| Q92911 | SC5A5 | 0.17 | Sodium/iodide cotransporter |
| P07098 | LIPF | 0.18 | Gastric triacylglycerol lipase |
| P20648 | ATP4A | 0.19 | Potassium-transporting ATPase alpha chain 1 |
| Q7Z3F1 | GPR155 | 0.19 | Integral membrane protein GPR155 |
| P17540 | CKMT2 | 0.19 | Creatine kinase S-type, mitochondrial |
| Q96NY7 | CLIC6 | 0.19 | Chloride intracellular channel protein 6 |
| P55087 | AQP4 | 0.19 | Aquaporin-4 |
| P27352 | CBLIF | 0.20 | Cobalamin binding intrinsic factor |
| Q9BY11 | PACSIN1 | 0.20 | Protein kinase C and casein kinase substrate in neurons protein 1 |
| P48052 | CPA2 | 0.20 | Carboxypeptidase A2 |
| P51164 | ATP4B | 0.21 | Potassium-transporting ATPase subunit beta |
| Q16772 | GSTA3 | 0.22 | Glutathione S-transferase A3 |
| Q7LBE3 | SLC26A9 | 0.22 | Solute carrier family 26 member 9 |
| P08263 | GSTA1 | 0.22 | Glutathione S-transferase A1 |
| P12277 | CKB | 0.23 | Creatine kinase B-type |
| A8TX70 | COL6A5 | 0.23 | Collagen alpha-5(VI) chain |
| Q99962 | SH3GL2 | 0.23 | Endophilin-A1 |
| P48735 | IDH2 | 0.23 | Isocitrate dehydrogenase [NADP], mitochondrial |
| Q96NR8 | RDH12 | 0.23 | Retinol dehydrogenase 12 |
| O43704 | SULT1B1 | 0.23 | Sulfotransferase 1B1 |
| P05413 | FABP3 | 0.24 | Fatty acid-binding protein, heart |
| Q16798 | ME3 | 0.24 | NADP-dependent malic enzyme, mitochondrial |
| P48764 | SLC9A3 | 0.24 | Sodium/hydrogen exchanger 3 |
| Q8TD20 | SLC2A12 | 0.25 | Solute carrier family 2, facilitated glucose transporter member 12 |
| P24298 | GPT | 0.25 | Alanine aminotransferase 1 |
| P04920 | SLC4A2 | 0.26 | Anion exchange protein 2 |
| Q8N3J6 | CADM2 | 0.27 | Cell adhesion molecule 2 |
| P55809 | OXCT1 | 0.27 | Succinyl-CoA:3-ketoacid coenzyme A transferase 1, mitochondrial |
| Q9GZV4 | EIF5A2 | 0.28 | Eukaryotic translation initiation factor 5A-2 |
| Q6P461 | ACSM6 | 0.28 | Acyl-coenzyme A synthetase ACSM6, mitochondrial |
| P01040 | CSTA | 0.28 | Cystatin-A |
| Q9H8M1 | COQ10B | 0.28 | Coenzyme Q-binding protein COQ10 homolog B, mitochondrial |
| Q99798 | ACO2 | 0.29 | Aconitate hydratase, mitochondrial |
| Q06520 | SULT2A1 | 0.30 | Sulfotransferase 2A1 |
| P15428 | HPGD | 0.30 | 15-hydroxyprostaglandin dehydrogenase [NAD(+)] |
| P02794 | FTH1 | 0.31 | Ferritin heavy chain |
| P50225 | SULT1A1 | 0.33 | Sulfotransferase 1A1 |
| Q9Y5U8 | MPC1 | 0.33 | Mitochondrial pyruvate carrier 1 |
| P11182 | DBT | 0.34 | Lipoamide acyltransferase component of branched-chain alpha-keto acid dehydrogenase complex, mitochondrial |
| Q96AN5 | TMEM143 | 0.34 | Transmembrane protein 143 |
| Q13825 | AUH | 0.34 | Methylglutaconyl-CoA hydratase, mitochondrial |
| Q96RQ3 | MCCC1 | 0.34 | Methylcrotonoyl-CoA carboxylase subunit alpha, mitochondrial |
| Q8WTR2 | DUSP19 | 0.34 | Dual specificity protein phosphatase 19 |
| Q8N3J5 | PPM1K | 0.35 | Protein phosphatase 1K, mitochondrial |
| P21810 | BGN | 0.35 | Biglycan |
| P22692 | IGFBP4 | 0.35 | Insulin-like growth factor-binding protein 4 |
| P21953 | BCKDHB | 0.35 | 2-oxoisovalerate dehydrogenase subunit beta, mitochondrial |
| Q13423 | NNT | 0.35 | NAD(P) transhydrogenase, mitochondrial |
| Q8N468 | MFSD4A | 0.36 | Major facilitator superfamily domain-containing protein 4A |
| O43920 | NDUFS5 | 0.36 | NADH dehydrogenase [ubiquinone] iron-sulfur protein 5 |
| Q16134 | ETFDH | 0.36 | Electron transfer flavoprotein-ubiquinone oxidoreductase, mitochondrial |
| P19404 | NDUFV2 | 0.36 | NADH dehydrogenase [ubiquinone] flavoprotein 2, mitochondrial |
| O75521 | ECI2 | 0.36 | Enoyl-CoA delta isomerase 2 |
| P12235 | SLC25A4 | 0.36 | ADP/ATP translocase 1 |
| O95563 | MPC2 | 0.37 | Mitochondrial pyruvate carrier 2 |
| O00483 | NDUFA4 | 0.37 | Cytochrome c oxidase subunit NDUFA4 |
| Q9UHQ9 | CYB5R1 | 0.37 | NADH-cytochrome b5 reductase 1 |
| P48047 | ATP5PO | 0.37 | ATP synthase subunit O, mitochondrial |
| Q9NXA8 | SIRT5 | 0.37 | NAD-dependent protein deacylase sirtuin-5, mitochondrial |
| P03928 | MT-ATP8 | 0.37 | ATP synthase protein 8 |
| P30038 | ALDH4A1 | 0.38 | Delta-1-pyrroline-5-carboxylate dehydrogenase, mitochondrial |
| P12694 | BCKDHA | 0.38 | 2-oxoisovalerate dehydrogenase subunit alpha, mitochondrial |
| Q9NUB1 | ACSS1 | 0.38 | Acetyl-coenzyme A synthetase 2-like, mitochondrial |
| P24539 | ATP5PB | 0.38 | ATP synthase F(0) complex subunit B1, mitochondrial |
| P10176 | COX8A | 0.38 | Cytochrome c oxidase subunit 8A, mitochondrial |
| Q6JQN1 | ACAD10 | 0.38 | Acyl-CoA dehydrogenase family member 10 |
| P31937 | HIBADH | 0.38 | 3-hydroxyisobutyrate dehydrogenase, mitochondrial |
| Q9NX14 | NDUFB11 | 0.39 | NADH dehydrogenase [ubiquinone] 1 beta subcomplex subunit 11, mitochondrial |
| P36542 | ATP5F1C | 0.39 | ATP synthase subunit gamma, mitochondrial |
| Q8NHY0 | B4GALNT2 | 0.39 | Beta-1,4 N-acetylgalactosaminyltransferase 2 |
| P21912 | SDHB | 0.39 | Succinate dehydrogenase [ubiquinone] iron-sulfur subunit, mitochondrial |
| O75489 | NDUFS3 | 0.39 | NADH dehydrogenase [ubiquinone] iron-sulfur protein 3, mitochondrial |
| Q8IWW8 | ADHFE1 | 0.39 | Hydroxyacid-oxoacid transhydrogenase, mitochondrial |
| Q9Y2R0 | COA3 | 0.39 | Cytochrome c oxidase assembly factor 3 homolog, mitochondrial |
| P00403 | MT-CO2 | 0.39 | Cytochrome c oxidase subunit 2 |
| P15954 | COX7C | 0.39 | Cytochrome c oxidase subunit 7C, mitochondrial |
| P17174 | GOT1 | 0.39 | Aspartate aminotransferase, cytoplasmic |
| P10606 | COX5B | 0.40 | Cytochrome c oxidase subunit 5B, mitochondrial |
| P00352 | ALDH1A1 | 0.40 | Aldehyde dehydrogenase 1A1 |
| Q8TD30 | GPT2 | 0.40 | Alanine aminotransferase 2 |
| Q02218 | OGDH | 0.40 | 2-oxoglutarate dehydrogenase complex component E1 |
| P16219 | ACADS | 0.40 | Short-chain specific acyl-CoA dehydrogenase, mitochondrial |
| Q86WU2 | LDHD | 0.40 | Probable D-lactate dehydrogenase, mitochondrial |
| P03915 | MT-ND5 | 0.40 | NADH-ubiquinone oxidoreductase chain 5 |
| P45954 | ACADSB | 0.40 | Short/branched chain specific acyl-CoA dehydrogenase, mitochondrial |
| P35900 | KRT20 | 0.40 | Keratin, type I cytoskeletal 20 |
| Q16698 | DECR1 | 0.41 | 2,4-dienoyl-CoA reductase [(3E)-enoyl-CoA-producing], mitochondrial |
| O75438 | NDUFB1 | 0.41 | NADH dehydrogenase [ubiquinone] 1 beta subcomplex subunit 1 |
| Q16718 | NDUFA5 | 0.41 | NADH dehydrogenase [ubiquinone] 1 alpha subcomplex subunit 5 |
| P03897 | MT-ND3 | 0.41 | NADH-ubiquinone oxidoreductase chain 3 |
| Q16795 | NDUFA9 | 0.41 | NADH dehydrogenase [ubiquinone] 1 alpha subcomplex subunit 9, mitochondrial |
| O43676 | NDUFB3 | 0.41 | NADH dehydrogenase [ubiquinone] 1 beta subcomplex subunit 3 |
| O95831 | AIFM1 | 0.41 | Apoptosis-inducing factor 1, mitochondrial |
| O75947 | ATP5PD | 0.42 | ATP synthase subunit d, mitochondrial |
| P14406 | COX7A2 | 0.42 | Cytochrome c oxidase subunit 7A2, mitochondrial |
| Q9ULD0 | OGDHL | 0.42 | 2-oxoglutarate dehydrogenase-like, mitochondrial |
| P11310 | ACADM | 0.42 | Medium-chain specific acyl-CoA dehydrogenase, mitochondrial |
| P31040 | SDHA | 0.42 | Succinate dehydrogenase [ubiquinone] flavoprotein subunit, mitochondrial |
| P56381 | ATP5F1E | 0.42 | ATP synthase subunit epsilon, mitochondrial |
| P13073 | COX4I1 | 0.42 | Cytochrome c oxidase subunit 4 isoform 1, mitochondrial |
| Q7L804 | RAB11FIP2 | 0.42 | Rab11 family-interacting protein 2 |
| Q9H0N5 | PCBD2 | 0.42 | Pterin-4-alpha-carbinolamine dehydratase 2 |
| P99999 | CYCS | 0.42 | Cytochrome c |
| P30405 | PPIF | 0.42 | Peptidyl-prolyl cis-trans isomerase F, mitochondrial |
| Q53S33 | BOLA3 | 0.42 | BolA-like protein 3 |
| Q96I99 | SUCLG2 | 0.43 | Succinate--CoA ligase [GDP-forming] subunit beta, mitochondrial |
| O76062 | TM7SF2 | 0.43 | Delta(14)-sterol reductase TM7SF2 |
| O75390 | CS | 0.43 | Citrate synthase, mitochondrial |
| Q9UIJ7 | AK3 | 0.44 | GTP:AMP phosphotransferase AK3, mitochondrial |
| O95822 | MLYCD | 0.44 | Malonyl-CoA decarboxylase, mitochondrial |
| P08559 | PDHA1 | 0.44 | Pyruvate dehydrogenase E1 component subunit alpha, somatic form, mitochondrial |
| Q8N142 | ADSS1 | 0.44 | Adenylosuccinate synthetase isozyme 1 |
| P30046 | DDT | 0.44 | D-dopachrome decarboxylase |
| P56134 | ATP5MF | 0.44 | ATP synthase subunit f, mitochondrial |
| P05141 | SLC25A5 | 0.44 | ADP/ATP translocase 2 |
| P30044 | PRDX5 | 0.44 | Peroxiredoxin-5, mitochondrial |
| Q9H0T7 | RAB17 | 0.45 | Ras-related protein Rab-17 |
| Q9UDX5 | MTFP1 | 0.45 | Mitochondrial fission process protein 1 |
| P07954 | FH | 0.45 | Fumarate hydratase, mitochondrial |
| P53597 | SUCLG1 | 0.45 | Succinate--CoA ligase [ADP/GDP-forming] subunit alpha, mitochondrial |
| Q9NPH0 | ACP6 | 0.45 | Lysophosphatidic acid phosphatase type 6 |
| P28331 | NDUFS1 | 0.46 | NADH-ubiquinone oxidoreductase 75 kDa subunit, mitochondrial |
| Q9NTX5 | ECHDC1 | 0.46 | Ethylmalonyl-CoA decarboxylase |
| MDH1 | MDH1 | 0.46 | Malate dehydrogenase, cytoplasmic |
| Q9HCC0 | MCCC2 | 0.46 | Methylcrotonoyl-CoA carboxylase beta chain, mitochondrial |
| Q8N442 | GUF1 | 0.46 | Translation factor GUF1, mitochondrial |
| O95182 | NDUFA7 | 0.46 | NADH dehydrogenase [ubiquinone] 1 alpha subcomplex subunit 7 |
| P07195 | LDHB | 0.46 | L-lactate dehydrogenase B chain |
| P47985 | UQCRFS1 | 0.46 | Cytochrome b-c1 complex subunit Rieske, mitochondrial |
| O75964 | ATP5MG | 0.46 | ATP synthase subunit g, mitochondrial |
| O95168 | NDUFB4 | 0.47 | NADH dehydrogenase [ubiquinone] 1 beta subcomplex subunit 4 |
| P05496 | ATP5MC1 | 0.47 | ATP synthase F(0) complex subunit C1, mitochondrial |
| Q9UI09 | NDUFA12 | 0.47 | NADH dehydrogenase [ubiquinone] 1 alpha subcomplex subunit 12 |
| O43674 | NDUFB5 | 0.47 | NADH dehydrogenase [ubiquinone] 1 beta subcomplex subunit 5, mitochondrial |
| Q96H55 | MYO19 | 0.47 | Unconventional myosin-XIX |
| O75306 | NDUFS2 | 0.47 | NADH dehydrogenase [ubiquinone] iron-sulfur protein 2, mitochondrial |
| Q8TB22 | SPATA20 | 0.47 | Spermatogenesis-associated protein 20 |
| Q16836 | HADH | 0.47 | Hydroxyacyl-coenzyme A dehydrogenase, mitochondrial |
| Q8N465 | D2HGDH | 0.47 | D-2-hydroxyglutarate dehydrogenase, mitochondrial |
| Q9P2M4 | TBC1D14 | 0.48 | TBC1 domain family member 14 |
| P15311 | EZR | 0.48 | Ezrin |
| P00505 | GOT2 | 0.48 | Aspartate aminotransferase, mitochondrial |
| O43772 | SLC25A20 | 0.48 | Mitochondrial carnitine/acylcarnitine carrier protein |
| Q9Y3B8 | REXO2 | 0.48 | Oligoribonuclease, mitochondrial |
| P07919 | UQCRH | 0.48 | Cytochrome b-c1 complex subunit 6, mitochondrial |
| Q5TGZ0 | MICOS10 | 0.48 | MICOS complex subunit MIC10 |
| Q96IX5 | ATP5MK | 0.48 | ATP synthase membrane subunit K, mitochondrial |
| P49821 | NDUFV1 | 0.48 | NADH dehydrogenase [ubiquinone] flavoprotein 1, mitochondrial |
| O75251 | NDUFS7 | 0.48 | NADH dehydrogenase [ubiquinone] iron-sulfur protein 7, mitochondrial |
| P22695 | UQCRC2 | 0.49 | Cytochrome b-c1 complex subunit 2, mitochondrial |
| Q8WVI0 | UQCC5 | 0.49 | Ubiquinol-cytochrome-c reductase complex assembly factor 5 |
| Q9UKU7 | ACAD8 | 0.49 | Isobutyryl-CoA dehydrogenase, mitochondrial |
| P00846 | MT-ATP6 | 0.49 | ATP synthase subunit a |
| P40939 | HADHA | 0.49 | Trifunctional enzyme subunit alpha, mitochondrial |
| P23434 | GCSH | 0.50 | Glycine cleavage system H protein, mitochondrial |
| Q9BV79 | MECR | 0.50 | Enoyl-[acyl-carrier-protein] reductase, mitochondrial |
| Q6IPR1 | ETFRF1 | 0.50 | Electron transfer flavoprotein regulatory factor 1 |
| Q5XKP0 | MICOS13 | 0.50 | MICOS complex subunit MIC13 |
| Q9Y394 | DHRS7 | 0.50 | Dehydrogenase/reductase SDR family member 7 |
| Q9HAV7 | GRPEL1 | 0.50 | GrpE protein homolog 1, mitochondrial |
| Q02338 | BDH1 | 0.50 | D-beta-hydroxybutyrate dehydrogenase, mitochondrial |
